# Phenome-Wide Associations of Polygenic Scores for Schizophrenia and Major Depression in 100,000 Chinese Adults

**DOI:** 10.1016/j.bpsgos.2025.100681

**Published:** 2025-12-22

**Authors:** Baihan Wang, Sam Morris, Hannah Fry, Andri Iona, Jonathan Clarke, Kuang Lin, Igor Pupko, Christiana Kartsonaki, Derrick A. Bennett, Yiping Chen, Huaidong Du, Ling Yang, Daniel Avery, Dan Schmidt-Valle, Shixian Feng, Dianjianyi Sun, Canqing Yu, Jun Lv, Pei Pei, Junshi Chen, Karoline Kuchenbaecker, Naomi R. Wray, Liming Li, Robin G. Walters, Zhengming Chen, Iona Y. Millwood, the China Kadoorie Biobank Collaborative Group

**Affiliations:** a Clinical Trial Service Unit, Nuffield Department of Population Health, University of Oxford, Oxford, United Kingdom; b Division of Psychiatry, University College London, London, United Kingdom; c Non-Communicable Diseases Prevention and Control Department, Henan Centre for Disease Control and Prevention, Zhengzhou, China; d Department of Epidemiology and Biostatistics, School of Public Health, Peking University, Beijing, China; e Peking University Center for Public Health and Epidemic Preparedness and Response, Peking University, Beijing, China; f Key Laboratory of Epidemiology of Major Diseases (Peking University), Ministry of Education, Beijing, China; g China National Center for Food Safety Risk Assessment, Beijing, China; h Department of Psychiatry, University of Oxford, Oxford, United Kingdom; i Institute for Molecular Bioscience, University of Queensland, Brisbane, Queensland, Australia

**Keywords:** Causal inference, Cross-population comparison, Depression, Phenome-wide association study, Polygenic score, Schizophrenia

## Abstract

**Background:**

China faces significant mental health challenges, with unique associations between mental disorders and other traits observed in its population.

**Methods:**

Based on summary statistics of existing genome-wide association studies in East Asian ancestry (EAS) and European ancestry (EUR) populations, we tested the associations of polygenic scores (PGSs) for schizophrenia (SCZ) and major depression (MD) with 254 phenotypes in 100,640 Chinese adults. We also conducted genetic correlation and Mendelian randomization analyses to assess the consistency of these associations across ancestries and infer causality.

**Results:**

The PGSs predicted SCZ (*R*^2^ = 2.63%–3.07%) and MD (*R*^2^ = 0.21%–0.71%) and were associated with various sociodemographic, lifestyle, and physical factors. Interestingly, based on summary statistics in the EAS population, the schizophrenia PGS was inversely associated with smoking initiation, and the MD PGS was inversely associated with body mass index. Across populations, opposing genetic correlations were observed between smoking initiation and SCZ (inverse in the EAS population, positive in the EUR population) and between body mass index and MD (inverse in the EAS population, positive in the EUR population). Univariable Mendelian randomization supported the causality of these relationships in the EUR population, but multivariable analyses suggested that pleiotropic effects on other related traits (e.g., cannabis use, unhealthy lifestyle) might have influenced the associations.

**Conclusions:**

Our study suggests the context specificity of relationships between mental disorders and other traits, highlighting a potential role of sociocultural factors.

## Plain Language Summary

The presentation of mental disorders in China can be distinct from that in other populations. Here, we calculated scores that indicate an individual’s genetic risk for schizophrenia and depression for ∼100,000 Chinese adults. We found that in China, people with a high schizophrenia score were less likely to smoke, and people with a high depression score had a lower BMI. These results contrast with findings in Western populations, possibly due to differences in sociocultural factors.

China accounts for 16% of the global burden of mental disorders (1), creating challenges for China’s national health care system. The number of disability-adjusted life years due to mental disorders in China has been rising steadily, with depressive disorders, anxiety disorders, and schizophrenia (SCZ) contributing to most of the burden (2).

Many mental disorders show significant heritability, with SCZ and major depression (MD) having around 80% and 30% heritability, respectively (3,4). Genetic correlations between the East Asian ancestry (EAS) population and the European ancestry (EUR) population have been estimated to be substantial (>0.9) for both SCZ and MD (5, 6, 7). Nevertheless, important differences still exist. For example, Chen *et al.* (8) reported larger genetic differences between EAS and EUR populations for SCZ-associated variants compared with variants randomly selected from the whole genome. Moreover, using Mendelian randomization (MR), O’Loughlin *et al.* (9) reported that lower body mass index (BMI) was causally associated with a higher risk of MD in the Chinese population, which was in a direction opposite to that of the causal association in the EUR population. This suggests that sociocultural factors potentially influence the relationships between mental disorders and other health-related traits (9).

Polygenic scores (PGSs) are the sum of trait-associated variants weighted by their effect sizes and broadly represent an individual’s genetic predisposition to a given trait (10). They have been found to be predictive of mental disorders in clinical and population-based studies (11). PGSs can also be used in phenome-wide association studies (PheWASs) to reveal associations between the genetic risk for mental disorders and a range of other traits across the human phenome (12). For example, previous PheWASs conducted in Western and EUR populations have reported associations of the PGS for SCZ or MD with other mental disorders, physical illnesses, behavioral traits, and brain structure measures (13, 14, 15, 16), suggesting shared genetic mechanisms and new modifiable risk factors.

Nevertheless, few PheWASs using the PGS for SCZ or MD have been conducted in the Chinese population. Such studies will help discover setting-specific associations, clarify disease etiology, and inform public health policies. Therefore, the aims of the current study are 1) to identify associations of the genetic risk for SCZ and MD with 254 phenotypes measured in 100,640 Chinese adults, 2) to examine the consistency of these associations in both EAS and EUR populations using genetic correlation analyses, and 3) to assess the causal relevance of these associations using univariable and multivariable MR.

## Methods and Materials

### Study Population

The CKB (China Kadoorie Biobank) is a prospective cohort study with >512,000 adults ages 30 to 79 years, recruited from 2004 to 2008 from 10 regions across urban and rural areas in China (17). Laptop-based questionnaire data and physical measurements were collected at baseline, with participants’ long-term health monitored via linkage with local death or disease registries, as well as the national health insurance systems. The current study is based on the follow-up data until Jan 1, 2019 (median = 12.36 [Q1–Q3: 11.20–13.27] years follow-up). Ethical approval was obtained from the Ethical Review Committee of the Chinese Centre for Disease Control and Prevention (CDC), Beijing, China (005/2004), and the Oxford Tropical Research Ethics Committee, University of Oxford, United Kingdom (025-04). All participants provided written informed consent.

### Genotyping

The current study included a total of 100,640 CKB participants, genotyped with a custom Affymetrix array (18). Of these, 23,518 were cases of cardiovascular disease or chronic obstructive pulmonary disease (COPD) selected for a nested case-control study, while the remaining 77,122 were randomly selected and population representative (18). Genotyped variants were prephased using SHAPEIT version 4.2 (SHAPEIT version 2.904 for chromosome X) (19) and uploaded to the TOPMed (20) and Westlake Biobank for Chinese (21) servers for imputation. Two sets of imputed data were merged by selecting the imputed genotype with a higher imputation INFO score for each variant.

### PGS Computation

We used PRS-CSx (22,23) to estimate the PGS weights for SCZ and MD based on 2 types of genome-wide association study (GWAS) summary statistics (24,25) (Table S1): one from GWASs conducted in EAS populations (excluding CKB data), including population- and setting-specific genetic associations, and the other from both GWASs in EAS populations and GWASs in EUR populations, which may improve overall predictive power (23). As a result, 4 different PGSs were computed for each participant: PGS-SCZ-EAS, PGS-SCZ-multi (EAS+EUR), PGS-MD-EAS, and PGS-MD-multi (EAS+EUR). We used samples from the EAS and EUR superpopulations in the 1000 Genomes Project (26) as linkage disequilibrium (LD) references. Only HapMap3 variants with a minor allele frequency (MAF) >0.01 in the relevant ancestry were included (27). We used PLINK (28) to apply the weights and generated PGSs for all 100,640 genotyped participants in the CKB. The PGSs were then standardized based on the means and SDs among the 77,122 population-representative CKB participants.

### Associations With SCZ and MD

We identified cases of SCZ based on hospitalization records at follow-up (ICD-10 code: F20). Cases of MD were identified based on both the Composite International Diagnostic Inventory-short form at baseline and hospitalization records at follow-up (ICD-10 codes: F32, F33, F34.1, and F38.1) (7,29). To maximize power and avoid potential biases, we identified cases from the overall dataset, while controls were only identified from the population-representative subset (Figure S1). With SCZ/MD as the outcome variable, we ran logistic regression analyses with the standardized PGS for SCZ/MD as the main explanatory variable, including age, age^2^, sex, study region, and the first 11 genomic principal components (PCs) as covariates to account for potential confounding and improve model fit. In particular, age^2^ was included to account for the nonlinear relationships between age and certain phenotypes, while the first 11 genomic PCs were found to be most informative of the population structure in the CKB (18).

### Phenome-Wide Association Studies

We included 254 phenotypes measured at baseline or during follow-up in a PheWAS. These included 67 baseline characteristics, with categorical variables of more than 2 levels being converted to binary variables, as described in Table S2. For phenotypes at follow-up, we used phecodes (version 1.2) (30) to capture incident diseases at follow-up, which are clinically meaningful disease categories based on ICD-10 codes. There were 187 3-digit phecodes with >100 cases in CKB genotyped participants. This resulted in ∼80% power to detect an odds ratio (OR) of 1.5 per SD higher PGS with an alpha = 0.001.

We applied logistic and linear regression models to assess the associations between PGSs and categorical and continuous phenotypes, respectively. Age, age^2^, sex, study region, and the first 11 genomic PCs were included as covariates. To maximize power and avoid potential biases, we identified cases of all disease-related phenotypes from the overall dataset, while controls were only identified from the population-representative subset (Figure S1). For each phecode, we excluded controls with related conditions defined by its phecode exclude range (30). Analyses of all other phenotypes were restricted to the population-representative participants. Multiple testing was corrected with a false discovery rate = .05, applied to the combined results of all 4 PGSs (254 × 4 = 1016 tests in total). The same procedure was repeated in sex-specific analyses for all phenotypes included in the overall analysis. For associations identified in the PheWAS, we performed sensitivity analyses by including household size, household income, ownership index, education level, BMI, smoking, and alcohol drinking as additional covariates.

### Genetic Correlations

For phenotypes that showed significant associations with PGSs in the PheWAS, we downloaded their GWAS summary statistics if they were publicly available in both EAS and EUR populations. For GWASs in the EAS population, we selected studies that were not conducted in the CKB or meta-analyses that included the CKB as only one of the many contributing cohorts (Table S1). All GWAS summary statistics were processed and harmonized using the GWASLab package (31). We used LD score regression (32) to compute the genetic correlations (*r*_g_) for all mental disorder–phenotype pairs within each ancestry. We performed additional analyses using Popcorn (33) to compute the genetic-effect correlations (ρ_ge_) across the EAS and EUR groups. All genetic correlation analyses were conducted with variants with MAF >0.01 and present in HapMap3 (27), based on EAS and EUR LD references from the 1000 Genomes Project (26).

### Mendelian Randomization

For mental disorder–phenotype pairs that showed associations in both PheWAS and genetic correlation analyses, we used MR to assess their causal relevance. There are 3 core assumptions of MR: 1) relevance, 2) independence, and 3) the exclusion restriction assumption.

We conducted bidirectional 2-sample MR using the TwoSampleMR (34) package based on publicly available GWAS summary statistics. To assess MR assumption 1, we calculated the mean *F* statistics for each exposure. To meet MR assumption 2, we performed MR with exposure and outcome GWASs from the same ancestry, separately for EAS and EUR populations, to avoid confounding due to ancestry. To remove correlated variants, we performed LD clumping with window = 10,000 kb and *r*^2^ = 0.001 based on EAS/EUR LD references from the 1000 Genomes Project (26). Due to the relatively small sample sizes of GWAS in the EAS population, we selected variants that reached suggestive genome-wide significance (*p* = 1 × 10^−5^) as genetic instruments in the main analyses (35) and performed sensitivity analyses with variants that reached genome-wide significance (*p* = 5 × 10^−8^). Genetic instruments in the EUR population were selected only if they reached genome-wide significance. We used 4 different methods to perform the 2-sample MR: inverse-variance weighting (main method) (35), MR-Egger (36), weighted median (37), and weighted mode (38).

To assess MR assumption 3, we further performed summary-level multivariable MR (39) using the TwoSampleMR (34) and MVMR (40) packages to account for potential pleiotropic effects on other phenotypes (i.e., BMI, smoking, educational attainment, and income) in the EUR population. These phenotypes were chosen as they were associated with SCZ/MD in our univariable MR analyses or identified as credible risk factors for SCZ/MD in previous prospective studies (41), and also had sufficiently powered GWASs. This was done by adding each of the extra phenotypes as an additional exposure. We first performed LD clumping for all pairs of exposures with window = 10,000 kb, *r*^2^ = 0.001, and *p* = 5 × 10^−8^, based on the EUR LD reference from the 1000 Genomes Project (26). Then, we estimated the pairwise covariances between each instrument and its corresponding pairs of exposures, based on the phenotypic correlations between exposures in the UK Biobank (Supplemental Note 2 and Table S3). We also estimated conditional *F* statistics to test instrument strength and *Q* statistics to test horizontal pleiotropy (39). For sensitivity analyses, we used *Q*-statistic minimization to reestimate beta, which is robust to weak instruments and pleiotropy (39). All MR analyses were conducted in R version 4.4.1 (42), and proxies with a minimum *r*^2^ = 0.8 were identified if genetic instruments were not present in the outcome.

## Results

### PGS Associations With SCZ and MD

Both PGS-SCZ-EAS and PGS-SCZ-multi were positively associated with the risk of SCZ (158 cases and 77,148 controls) (Table 1). PGS-SCZ-multi explained slightly more variance in SCZ (*R*^2^ = 3.07%) on the liability scale compared with PGS-SCZ-EAS (*R*^2^ = 2.63%). Similarly, both PGS-MD-EAS and PGS-MD-multi were positively associated with the risk of MD (906 cases and 77,321 controls) (Table 1), but PGS-MD-multi explained more variance on the liability scale compared with PGS-MD-EAS (*R*^2^ = 0.71% vs. 0.21%). The distributions of PGSs between cases and controls as well as ORs per PGS quartile are shown in Figures S1 and S2. All PGSs were associated with their corresponding phenotypes in sex-specific analyses (Table S4 and Figures S2–S7).

**Table 1:** Associations Between PGSs for SCZ or MD and Their Corresponding Phenotypes in the China Kadoorie Biobank

Mental Disorder	Cases	Controls	GWAS Source	OR per SD Higher PGS (95% CI)	*p*	*R*^2^ on Liability Scale
SCZ	158	77,148	EAS	1.63 (1.39–1.92)	4.77 × 10^−9^	2.63%
			Multi (EAS + EUR)	1.67 (1.42–1.96)	5.44 × 10^−10^	3.07%
MD	906	77,321	EAS	1.13 (1.06–1.20)	3.79 × 10^−4^	0.21%
			Multi (EAS + EUR)	1.26 (1.18–1.34)	1.27 × 10^−11^	0.71%

Cases of mental disorders were identified from the overall dataset, while controls were identified from the population-representative subset. Two types of PGSs were assessed for each mental disorder, one based on a GWAS in the EAS population and one based on a GWAS in both EAS and EUR populations. OR is per 1 SD higher PGS.

EAS, East Asian ancestry; EUR, European ancestry; GWAS, genome-wide association study; MD, major depression; OR, odds ratio; PGS, polygenic score; SCZ, schizophrenia.

### Phenome-Wide Associations

**Figure 1:**
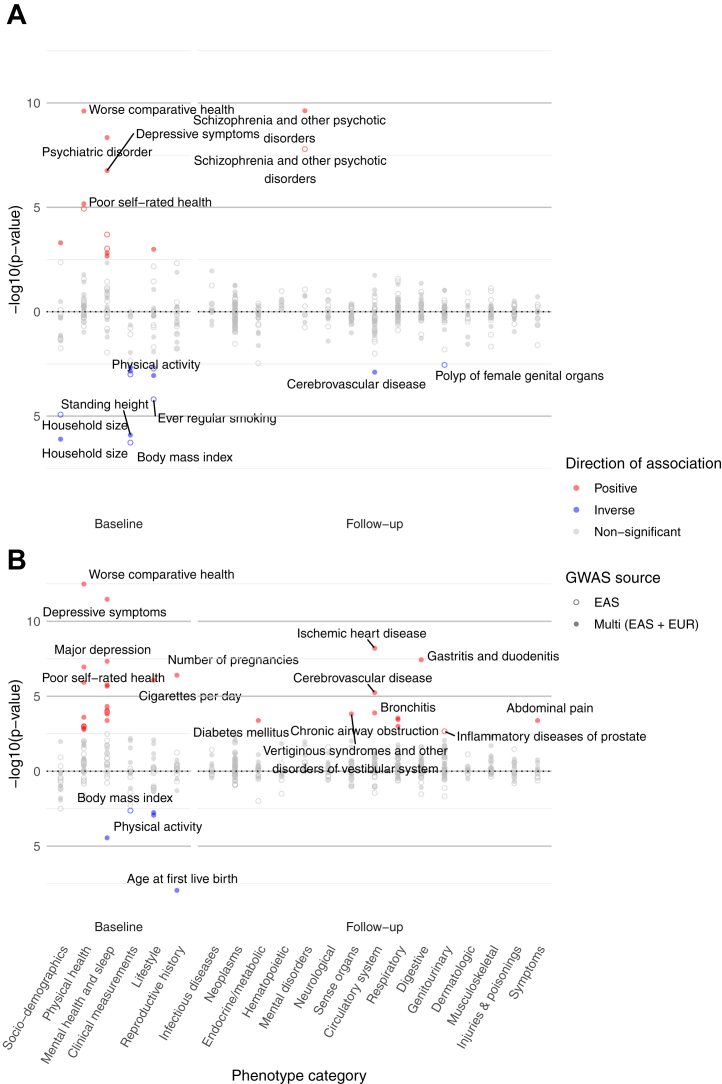
Phenome-wide associations with polygenic scores for schizophrenia and major depression in the China Kadoorie Biobank. **(A)** Results of polygenic scores for schizophrenia. **(B)** Results of polygenic scores for major depression. A total of 254 phenotypes (67 baseline measures and 187 phecodes recorded during follow-up) were tested. Phenotypes only present in 1 sex are also included and shown here. The shape of dots indicates the GWAS source, while the color of dots indicates the direction of association. Results were corrected for multiple testing with a false discovery rate = .05. The top 2 most significant associations in each phenotype category are labeled. EAS, East Asian ancestry; EUR, European ancestry; GWAS, genome-wide association study.

After correction for multiple testing (Figure 1), both PGS-SCZ-EAS and PGS-SCZ-multi were positively associated with a higher risk of psychotic disorders (phecode: 295) during follow-up (*p* < 3 × 10^−6^). At baseline, higher PGS-SCZ-EAS and PGS-SCZ-multi were both associated with self-reported psychiatric disorder and depressive symptoms, as well as worse subjective health, lower household size, and lower sitting height (*p* < .002). A higher PGS-SCZ-EAS was associated with lower BMI (beta per SD higher PGS = −0.06; 95% CI, −0.09 to −0.04; *p* = 5.37 × 10^−7^) and lower odds of ever regular smoking (OR = 0.95; 95% CI, 0.93 to 0.98; *p* = 6.37 × 10^−5^) at baseline, while directionally consistent but nonsignificant associations were found for PGS-SCZ-multi. In contrast, higher PGS-SCZ-multi was associated with more cigarettes smoked per day, higher levels of education, lower standing height, and lower physical activity (*p* < .002). Additionally, we observed inverse associations between PGS-SCZ-multi and cerebrovascular disease and between PGS-SCZ-EAS and female genital organ polyps at follow-up (*p* < .003), although the associations were attenuated after adjusting for additional covariates (Supplemental Data 1). Sex-specific PheWASs showed generally consistent results with the sex-combined analyses (Figures S8 and S9; Supplemental Data 1).

After correction for multiple testing (Figure 1), higher PGS-MD-EAS and PGS-MD-multi were associated with higher risks of self-reported depressive symptoms, as well as worse subjective health at baseline (*p* < 2 × 10^−4^). PGS-MD-EAS also showed a unique inverse association with BMI (β = −0.04; 95% CI, −0.06 to −0.01; *p* = .002), which was instead positively associated with PGS-MD-multi at nominal significance. Conversely, PGS-MD-multi yielded more associations in general compared with PGS-MD-EAS. At baseline, a higher PGS-MD-multi was associated with more cigarettes smoked per day, shorter and worse sleep, lower physical activity, younger age at first birth, and higher number of pregnancies (*p* < .002). At follow-up, a higher PGS-MD-multi was also associated with higher risks of a range of disorders and symptoms, such as anxiety and neurasthenia at baseline, as well as ischemic heart disease, gastritis and duodenitis, cerebrovascular disease, chronic airway obstruction, and diabetes mellitus (*p* < 4 × 10^−4^). Sex-specific PheWASs yielded results generally consistent with the sex-combined analyses (Figures S8 and S9; Supplemental Data 1).

### Genetic Correlations

**Figure 2:**
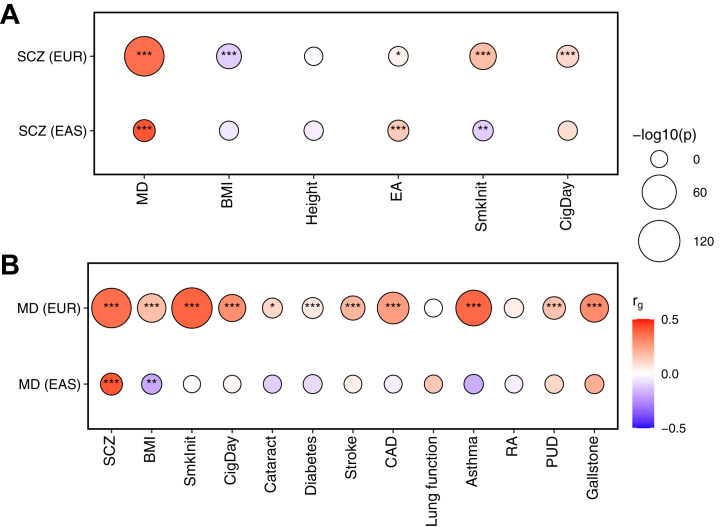
Genetic correlations between SCZ or MD and related phenotypes. **(A)** Genetic correlations with SCZ. **(B)** Genetic correlations with MD. Genetic correlations (*r*_g_) were tested by linkage disequilibrium score regression, using GWASs on SCZ/MD and phenotypes within the same ancestry. Mental disorder–phenotype pairs that were significant in the phenome-wide association analysis were tested here. Only phenotypes with publicly available GWASs in both EAS and EUR populations were included. The color of the circles indicates the correlation coefficient. The size of the circles is scaled to −log_10_(*p* value). ∗*p* < .05, ∗∗*p* < .01, ∗∗∗*p* < .001. BMI, body mass index; CAD, coronary artery disease; CigDay, cigarettes per day; EA, educational attainment; EAS, East Asian ancestry; EUR, European ancestry; GWAS, genome-wide association study; MD, major depression; PUD, peptic ulcer disease; RA, rheumatoid arthritis; SCZ, schizophrenia; SmkInit, smoking initiation.

As shown in Figure 2 and Table S5, there were positive genetic correlations between SCZ and MD in both EAS and EUR populations (*p* < .001). SCZ had an inverse genetic correlation with smoking initiation in the EAS (*r*_g_ = −0.10; 95% CI, −0.18 to −0.03; *p* = .005) but a positive genetic correlation with smoking initiation in the EUR (*r*_g_ = 0.17; 95% CI, 0.13 to 0.21; *p* = 7.01 × 10^−19^) populations. There was an inverse genetic correlation between MD and BMI (*r*_g_ = −0.19; 95% CI, −0.33 to −0.05; *p* = .009). In contrast, in the EUR population, MD showed a positive genetic correlation with BMI (*r*_g_ = 0.17; 95% CI, 0.14 to 0.20; *p* = 2.16 × 10^−27^). Cross-ancestry analyses showed positive genetic correlations between SCZ and MD (*p* < .05) (Figure S10 and Table S6).

### Mendelian Randomization

**Figure 3:**
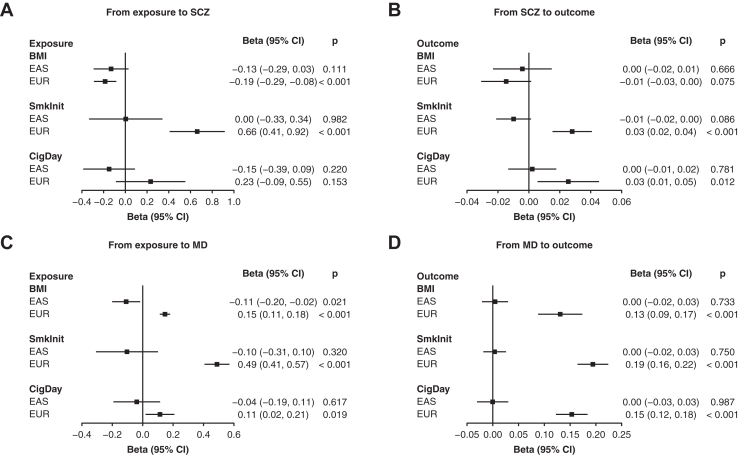
Bidirectional Mendelian randomization between SCZ or MD and related phenotypes. **(A)** From exposure to SCZ. **(B)** From SCZ to outcome. **(C)** From exposure to MD. **(D)** From MD to outcome. Results shown here are based on estimates from the inverse variance-weighted method. Genetic instruments were selected based on *p* < 1 × 10^−5^ in the EAS and *p* < 5 × 10^−8^ in the EUR populations after clumping. For continuous phenotypes, beta represents the change in the outcome per unit higher in the exposure. For binary phenotypes, such changes in the exposure/outcome are on the log-odds scale. BMI, body mass index; CigDay, cigarettes per day; EAS, East Asian ancestry; EUR, European ancestry; MD, major depression; SCZ, schizophrenia; SmkInit, smoking initiation.

Genetic instruments for all exposures in MR had a mean *F* statistic >20 (Supplemental Data 2). As shown in Figure 3, in the EUR population, genetically predicted BMI was inversely associated with SCZ (β = −0.19; 95% CI, −0.29 to −0.08; *p* < .001), while a positive bidirectional association was found between smoking initiation and SCZ (*p* < .001). In the EUR population, we also found positive bidirectional associations between BMI and MD (*p* < .001), as well as between smoking initiation and MD (*p* < .001). In contrast, we found an inverse association between genetically predicted BMI and MD (β = −0.11; 95% CI, −0.20 to −0.02; *p* = .021) (Figure 3). However, this association was no longer significant in our sensitivity analyses with alternative MR methods or with a more stringent *p*-value threshold (Supplemental Data 2 and Figure S11). Sensitivity analyses using other MR methods showed generally consistent results in the EUR population (Supplemental Data 2).

**Figure 4:**
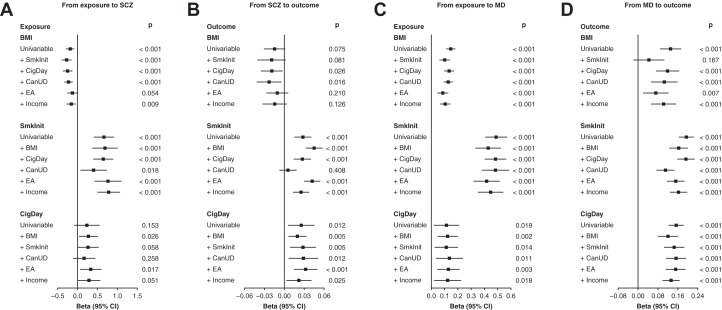
Multivariable Mendelian randomization between SCZ or MD and other phenotypes in the European ancestry population. **(A)** From exposure to SCZ. **(B)** From SCZ to outcome. **(C)** From exposure to MD. **(D)** From MD to outcome. All multivariable Mendelian randomization analyses were based on genome-wide association study summary statistics in the European ancestry population with the inverse variance-weighted method. Genetic instruments were selected based on *p* < 5 × 10^−8^ after clumping. An additional phenotype was added to the univariable model to assess its influence on the association each time. For continuous phenotypes, beta represents the change in the outcome per unit higher in the exposure. For binary phenotypes, such changes in the exposure/outcome are on the log-odds scale. Income represents income factor based on a multivariate analysis of individual, occupational, household, and parental incomes. BMI, body mass index; CanUD, cannabis use disorder; CigDay, cigarettes per day; EA, educational attainment; MD, major depression; SCZ, schizophrenia; SmkInit, smoking initiation.

Compared with univariable MR results, adding educational attainment as an additional exposure in multivariable MR attenuated the causal relationship of BMI with SCZ (Figure 4 and Supplemental Data 3). We also found that the bidirectional association between smoking initiation and SCZ was attenuated by adding cannabis use disorder as an additional exposure. Additionally, the causal relevance of MD to BMI was attenuated when including smoking initiation as an additional exposure. The conditional *F* statistics were >10 for most exposures, but all models had a *p* < .05 for their *Q* statistics. In the sensitivity analyses, *Q*-statistic minimization shifted most of the beta estimates closer to 0 (Supplemental Data 3). It is worth noting that because SCZ and MD could be viewed as dichotomizations of their underlying continuous psychopathology, the betas in our MR are better viewed as test statistics for causal inference on SCZ and MD liability instead of precise causal effect estimates (43).

## Discussion

This is the first study to systematically investigate the PheWASs of PGSs for SCZ and MD in the Chinese population. Both PGSs were associated with their corresponding mental disorders, as well as with a range of other phenotypes. In particular, we identified population-specific inverse associations between the genetic susceptibility for SCZ and smoking initiation and between the genetic susceptibility for MD and BMI. Genetic correlation analyses revealed opposing findings for these associations in EAS and EUR populations, although MR only supported causal relationships in the EUR population. Further multivariable MR analyses suggested that such causality in the EUR population might be accounted for by pleiotropic effects on other related traits.

Many of our PheWAS results in this Chinese population replicated previous findings in Western populations. For example, the PGSs for SCZ and MD were associated with various mental health–related phenotypes in our study, consistent with genetic associations that have been reported in the EUR population (44). Moreover, we found that PGS-SCZ was inversely associated with BMI and positively associated with education level, replicating previous findings in the EUR population (45,46), although multivariable MR indicated that this relationship might be influenced by pleiotropic effects on educational attainment. Additionally, we found that a higher PGS-MD-multi was associated with a younger age at first birth and a higher number of pregnancies in Chinese women, consistent with previous observational findings in the U.S. population (47,48). PGS-MD-multi was also associated with the risks of several physical illnesses at follow-up in the CKB, supporting previous observational associations of MD with ischemic heart disease (49), stroke (50), COPD (51), and diabetes (52,53).

In the PheWAS, PGS-SCZ-EAS was inversely associated with smoking initiation, which was supported by our genetic correlation analyses in the EAS population, although our MR analyses did not support a causal relationship in the EAS population. Conversely, we found a positive association between smoking initiation and SCZ in both our genetic correlation and MR analyses in the EUR population, consistent with previous findings in the EUR population (54,55). This difference between populations has also been reported in observational studies; while SCZ has been associated with higher rates of smoking in Western populations (55), studies in China have reported a similar prevalence of smoking in people with SCZ and the general population (56,57). Such differences may be explained by differences in smoking behavior, as China has a higher proportion of current smokers (24.3%) than many Western countries (58), such as the United Kingdom (11.9%) (59). Because smoking in China is often accepted as a social activity (60), people with a higher genetic risk for SCZ may also be less engaged in such behaviors. Cannabis use is another possible explanation for this difference, as it is a strong risk factor for SCZ (61) and often co-occurs with cigarette smoking in Western populations (62). In contrast, very few people use cannabis in China due to strict regulations, so the relationship between smoking and SCZ would not be influenced by this factor. In our multivariable MR analysis in the EUR population, adding cannabis use disorder as an additional exposure attenuated the association between smoking initiation and SCZ, suggesting potential pleiotropic effects in this population.

In the PheWAS, we observed a unique inverse association between PGS-MD-EAS and BMI, as well as an inverse genetic correlation between the phenotypes. Conversely, a positive genetic correlation between BMI and MD in the EUR population was observed in the current and previous studies (63). Further MR analyses revealed a positive association between genetically predicted BMI and MD in the EUR population but an inverse association in the EAS population, although the latter was not significant in our sensitivity analyses. The results are generally consistent with the inverse association between genetically predicted BMI and MD in the EAS population reported by O’Loughlin *et al.* (9), while the current study involved more cases of MD by including cases identified at follow-up and adopted a stricter definition of controls by limiting the analysis to the population-representative subset. Sociocultural factors may explain this difference between populations: It has been hypothesized that higher BMI is viewed as a sign of wealth and health among middle-age and older adults in China, but people with higher BMI face more weight-based discrimination in Western societies (64). Additionally, the maladaptive coping explanation postulates that people with MD more often engage in unhealthy behaviors associated with weight gain (65), and such coping strategies may more commonly be adopted in Western societies (66). We hypothesize that smoking is potentially one such unhealthy behavior, which was supported by our MVMR analyses in the EUR population. It is worth noting that although smoking was previously often associated with lower BMI, a recent study reported that genetically predicted smoking initiation was positively associated with obesity-related traits among individuals ages 40 to 69 years, supporting a potential long-term adverse effect of smoking on BMI (67). Finally, medication usage is another important factor to consider, as antidepressants are commonly associated with weight gain, but the income-adjusted consumption of psychotropic drugs in China was only 3% of that in North America in 2019 (68). We were unable to explore this further due to the lack of medication use data in the CKB, but future research on this topic in China will be beneficial.

The current study has limitations. First, we did not include PGSs for anxiety disorders or bipolar disorder due to the lack of well-powered GWASs in the EAS population at the time of the study or limited cases in the CKB. Because the CKB follow-up data came from hospitalization and death records, a significant proportion of outpatient SCZ/MD cases might also have been missed, thus limiting the *R*^2^ explained by PGSs. Nevertheless, all PGSs tested here were strongly associated with SCZ and MD, supporting their validity in the PheWAS. Second, we adopted univariate linear and logistic regression models in the PheWAS. Although this provides a comprehensive overview of individual PGS-phenotype associations, future studies should also consider multivariable and multivariate models to explore the patterns of associations among multiple phenotypes. Third, some exposures (e.g., cannabis use disorder) had a conditional *F* statistic <10 in the multivariable MR analyses, indicating potential weak instrument bias. However, because *Q*-statistic minimization in the sensitivity analyses generally shifted the betas toward the null, we believe that the conclusions drawn from the multivariable MR are still valid. Fourth, some of our PheWAS findings require further explanation, such as the inverse associations of PGS-SCZ with cerebrovascular disease and female genital organ polyps. It is possible that such associations were mediated through other lifestyle and physical factors, considering the inverse associations of PGS-SCZ with smoking initiation and BMI, as well as the attenuation of these associations in our sensitivity analyses with additional covariates. The association with female genital organ polyps may also be explained by the observation of estrogen deficiency in SCZ (69), although more research is needed to replicate this finding. Finally, it is worth highlighting that other factors, such as differences in case definition, population history, allele frequency, and LD structure, may also explain the difference in genetic associations between populations. Although this is beyond the scope of the current study, other studies have identified specific SCZ- and MD-associated loci that showed low transferability between EAS and EUR populations (5,6). Further research investigating these low-transferability loci will be beneficial to clarify their influence on the relationship between mental disorders and other phenotypes.

### Conclusions

The current study found that the PGSs for SCZ and MD were associated with a range of phenotypes among ∼100,000 Chinese adults, suggesting their shared genetic architectures. The distinct associations of smoking with SCZ and BMI with MD across different populations highlight the important role that sociocultural factors may play in those relationships. More research in diverse populations is still needed to clarify the context specificity of mental disorders and their links with other health-related traits, and it remains to be answered whether interventions promoting smoking cessation and weight loss would be effective in reducing SCZ/MD risk in diverse populations. Policymakers should also be aware of such differences to develop better mental health strategies tailored for specific populations.

## Supplementary Material

Supplemental Notes 1–2, Tables S1–S6, and Figures S1–S11

Supplemental Data 3

Key Resources Table
