# Supplementary material for: Phenome-Wide Associations of Polygenic Scores for Schizophrenia and Major Depression in 100,000 Chinese Adults
Source: Biol Psychiatry Glob Open Sci. 2025 Dec 22;6(2):100681. doi: 10.1016/j.bpsgos.2025.100681 (PMC12907099; doi:10.1016/j.bpsgos.2025.100681)
Supplement: Supplemental Notes 1–2, Tables S1–S6, and Figures S1–S11 [file mmc1.pdf]

SUPPLEMENTARY INFORMATION

Phenome-Wide Associations of Polygenic Scores for Schizophrenia and Major Depression in 100,000 Chinese Adults

Wang *et al.*

Contents

Supplementary Note 1. Members of CKB Collaborative Group 3

Supplementary Note 2. Estimation of covariance matrix in multivariable Mendelian Randomisation. 4

Supplementary Table 1. GWAS summary statistics used in analyses. 5

Supplementary Table 2. Description of phenotypes at baseline used in PheWAS in CKB. 6

Supplementary Table 3. Phenotypic correlations between mental disorders and phenotypes in the UK Biobank. 11

Supplementary Table 4. Associations between polygenic scores for schizophrenia/major depression and their corresponding phenotypes in CKB by sex. 12

Supplementary Table 5. Within-ancestry genetic correlations between mental disorders and other phenotypes. 13

Supplementary Table 6. Cross-ancestry genetic correlations between mental disorders and other phenotypes. 15

Supplementary Figure 1. Selection of cases and controls in PheWAS. 16

Supplementary Figure 2. Performance of schizophrenia polygenic scores in CKB. . 17

Supplementary Figure 3. Performance of major depression polygenic scores in CKB. 18

Supplementary Figure 4. Performance of schizophrenia polygenic scores among females in CKB. 19

Supplementary Figure 5. Performance of major depression polygenic scores among females in CKB. 20

Supplementary Figure 6. Performance of schizophrenia polygenic scores among males in CKB. 21

Supplementary Figure 7. Performance of major depression polygenic scores among males in CKB. 22

Supplementary Figure 8. Phenome-wide associations with polygenic scores for schizophrenia and major depression among females in CKB. 23

Supplementary Figure 9. Phenome-wide associations with polygenic scores for schizophrenia and major depression among males in CKB. 24

Supplementary Figure 10. Cross-ancestry genetic correlations between schizophrenia/major depression and related phenotypes. 25

Supplementary Figure 11. Bi-directional Mendelian Randomisation between schizophrenia/major depression and other phenotypes with a more stringent threshold. 26

Supplementary Note 1. Members of CKB Collaborative Group

International Steering Committee: Junshi Chen, Zhengming Chen (PI), Robert Clarke, Rory Collins, Liming Li (PI), Jun Lv, Richard Peto, Robin Walters.

International Co-ordinating Centre, Oxford: Daniel Avery, Maxim Barnard, Derrick Bennett, Ruth Boxall, Ka Hung Chan, Yiping Chen, Zhengming Chen, Charlotte Clarke, Jonathan Clarke; Robert Clarke, Huaidong Du, Ahmed Edris Mohamed, Hannah Fry, Simon Gilbert, Prapthi Harish, Pek Kei Im, Andri Iona, Maria Kakkoura, Christiana Kartsonaki, Kshitij Kolhe, Hubert Lam, Kuang Lin, James Liu, Mohsen Mazidi, Iona Millwood, Sam Morris, Qunhua Nie, Alfred Pozarickij, Maryam Rahmati, Paul Ryder, Dan Schmidt, Becky Stevens, Iain Turnbull, Robin Walters, Baihan Wang, Lin Wang, Neil Wright, Ling Yang, Xiaoming Yang, Pang Yao.

National Co-ordinating Centre, Beijing: Xiao Han, Can Hou, Qingmei Xia, Chao Liu, Jun Lv, Pei Pei, Dianjianyi Sun, Canqing Yu, Lang Pan.

10 Regional Co-ordinating Centres:

Qingdao CDC: Zengchang Pang, Ruqin Gao, Shanpeng Li, Haiping Duan, Shaojie Wang, Yongmei Liu, Ranran Du, Yajing Zang, Liang Cheng, Xiaocao Tian, Hua Zhang, Yaoming Zhai, Feng Ning, Xiaohui Sun, Feifei Li. **Licang CDC:** Silu Lv, Junzheng Wang, Wei Hou. **Heilongjiang Provincial CDC:** Wei Sun, Shichun Yan, Xiaoming Cui. **Nangang CDC:** Chi Wang, Zhenyuan Wu, Yanjie Li, Quan Kang. **Hainan Provincial CDC:** Huiming Luo, Tingting Ou. **Meilan CDC:** Xiangyang Zheng, Zhendong Guo, Shukuan Wu, Yilei Li, Huimei Li. **Jiangsu Provincial CDC:** Ming Wu, Yonglin Zhou, Jinyi Zhou, Ran Tao, Jie Yang, Jian Su. **Suzhou CDC:** Fang Liu, Jun Zhang, Yihe Hu, Yan Lu, Liangcai Ma, Aiyu Tang, Shuo Zhang, Jianrong Jin, Jingchao Liu. **Guangxi Provincial CDC:** Mei Lin, Zhenzhen Lu. **Liuzhou CDC:** Lifang Zhou, Changping Xie, Jian Lan, Tingping Zhu, Yun Liu, Liuping Wei, Liyuan Zhou, Ningyu Chen, Yulu Qin, Sisi Wang. **Sichuan Provincial CDC:** Xianping Wu, Ningmei Zhang, Xiaofang Chen, Xiaoyu Chang. **Pengzhou CDC:** Mingqiang Yuan, Xia Wu, Xiaofang Chen, Wei Jiang, Jiaqiu Liu, Qiang Sun. **Gansu Provincial CDC:** Faqing Chen, Xiaolan Ren, Caixia Dong. **Maiji CDC:** Hui Zhang, Enke Mao, Xiaoping Wang, Tao Wang, Xi zhang. **Henan Provincial CDC:** Kai Kang, Shixian Feng, Huizi Tian, Lei Fan. **Huixian CDC:** XiaoLin Li, Huarong Sun, Pan He, Xukui Zhang. **Zhejiang Provincial CDC:** Min Yu, Ruying Hu, Hao Wang. **Tongxiang CDC**: Xiaoyi Zhang, Yuan Cao, Kaixu Xie, Lingli Chen, Dun Shen. **Hunan Provincial CDC:** Xiaojun Li, Donghui Jin, Li Yin, Huilin Liu, Zhongxi Fu. **Liuyang CDC:** Xin Xu, Hao Zhang, Jianwei Chen, Yuan Peng, Libo Zhang, Chan Qu.

Supplementary Note 2. Estimation of covariance matrix in multivariable Mendelian Randomisation.

A covariance matrix containing pairwise covariances between an instrument and pairs of exposures is required for causal effect estimation and sensitivity analyses in multivariable MR. This matrix can be estimated based on the phenotypic correlations between exposures using the MVMR package in R.

We used the UK Biobank data to calculate the pairwise phenotypic correlations between BMI, smoking initiation (ex- + current smokers), cigarettes per day (among current smokers), heavy cannabis use (lifetime cannabis use > 100 times), age at completion of full-time education, average annual household income, schizophrenia, and major depression. As many exposures were not continuous, we used Spearman's rank-based correlation to compute the correlation coefficient rho. Since very few people in the UKB had cannabis use disorder, we used heavy cannabis use as a proxy. Schizophrenia and major depression were defined based on self-reported diagnosis at baseline, as well as hospitalisation and death records at follow-up. All analyses were restricted to participants with self-reported White ethnicity. The correlation coefficients are shown in Supplementary Table 5.

Supplementary Table 1. GWAS summary statistics used in analyses.

Phenotype	Ancestry	Source	First author (year)	PMID/URL
Schizophrenia	EAS	PGC	Trubetskoy (2022)	35396580
Schizophrenia	EUR	PGC	Trubetskoy (2022)	35396580
Major depression	EAS	PGC	Meng (2024)	38177345
Major depression	EUR	PGC	Adams (2025)	39814019
Body mass index	EAS	BBJ	Akiyama (2017)	28892062
Body mass index	EUR	GIANT	Yengo (2018)	30124842
Height	EAS	GIANT	Yengo (2022)	36224396
Height	EUR	GIANT	Yengo (2022)	36224396
Educational attainment	EUR	SSGAC	Okbay (2022)	35361970
Educational attainment	EAS	Meta-analysis	Chen (2024)	38182883
Smoking initiation	EAS	GSCAN	Saunders (2022)	36477530
Smoking initiation	EUR	GSCAN	Saunders (2022)	36477530
Cigarettes per day	EAS	GSCAN	Saunders (2022)	36477530
Cigarettes per day	EUR	GSCAN	Saunders (2022)	36477530
Cataract	EAS	BBJ	Sakaue (2021)	34594039
Cataract	EUR	UKB	The Neale Lab (2018)	http://www.nealelab.is/uk-biobank/
Diabetes	EAS	Meta-analysis	Suzuki (2024)	38374256
Diabetes	EUR	Meta-analysis	Suzuki (2024)	38374256
Stroke	EAS	GIGASTROKE	Mishra (2022)	36180795
Stroke	EUR	GIGASTROKE	Mishra (2022)	36180795
Coronary artery disease	EAS	BBJ	Koyama (2020)	33020668
Coronary artery disease	EUR	CARDIoGRAMplusC4D	Aragam (2022)	36474045
Lung function (FEV1/FVC)	EAS	TWB	Chen (2023)	38116116
Lung function (FEV1/FVC)	EUR	Meta-analysis	Shrine (2023)	36914875
Asthma	EAS	GBMI	Tsuo (2022)	36778051
Asthma	EUR	GBMI	Tsuo (2022)	36778051
Rheumatoid arthritis	EAS	Meta-analysis	Ishigaki (2022)	36333501
Rheumatoid arthritis	EUR	Meta-analysis	Ishigaki (2022)	36333501
Pectic ulcer disease	EAS	Meta-analysis	He (2023)	38036781
Pectic ulcer disease	EUR	UKB	Wu (2021)	33608531
Gallstone	EAS	BBJ	Sakaue (2021)	34594039
Gallstone	EUR	Meta-analysis	Fairfield (2022)	34651315
Cannabis use disorder	EUR	MVP	Levery (2023)	37985822
Income	EUR	Meta-analysis	Kweon (2025)	39875632

Note. EAS: East Asian ancestry population. EUR: European ancestry population. BBJ: Biobank Japan. UKB: UK Biobank. TWB: Taiwan Biobank.

Supplementary Table 2. Description of phenotypes at baseline used in PheWAS in CKB.

Group	Phenotype	Description
Socio-demographics	Urban area	Is the region the participant lives in urban, compared to rural?
	Married	Is the participant married, compared to widowed, separated/divorced, and never married?
	Employed	Is the participant employed (employed in a specific industry; self-employed), compared to unemployed (retired; housewife/ husband; unemployed; other or not stated)?
	> 9 years education	Does the participant have greater than 9 years of education (high school; technical school or college; university), compared to less than 9 years of education (middle school; primary school; no formal education)
	Household size	How many people live in the participant's household?
	Number of children	How many children does the participant have?
	Ownership index	Derived by aggregating the following binary variables for each participant: possession of health cover; home ownership; access to private sanitation facilities; access to a motor vehicle; access to a phone; engagement in recent leisure travel.
	Household income >= 20000	Is the participant's income ≥ 20,000 yuan annually, compared to < 20,000 yuan annually?
Mental health and sleep	Major depression	In the past year, has the participant had major depression? Yes, if the participant reports at least three of the seven symptoms in CIDI-SF-A, including weight or appetite change, sleeping problems, psychomotor changes, fatigue, concentration problems, feelings of guilt or worthlessness and thoughts of suicide.
	Depressive symptoms	In the past year, has the participant had at least one of the four depressive symptoms, including feeling sad/depressed, feeling worthless and useless, loss of interest, and loss of appetite? Screening questions for major depression in CIDI-SF. If yes, participant completes CIDI-SF-A.
	Generalised anxiety disorder	In the past year, has the participant had generalised anxiety disorder in the past year? Yes, if the participant meets the following three conditions in CIDI-SF-B, including (1) the anxious period was stronger than in other people, lasted more days, and involved worrying about more than one thing; (2) had difficulty controlling the worries; (3) had at least three physiological symptoms
	Continuous anxiety	In the past year, has the participant had a period lasting one month or longer in the past year, when most of the time they felt worried, tense, or anxious, and it interfered with their life? Screening question for generalised anxiety disorder in CIDI-SF. If yes, participant completes CIDI-SF-B.
	Continuous pain	In the past year, has the participant experienced a pain or discomfort in their body lasting >=3 months that interfered with their life? (question in CIDI-SF)
	Panic attacks	In the past year, has the participant experienced a spell or an attack when all of a sudden they felt frightened, anxious, or very uneasy? (question in CIDI-SF)
	Phobia symptoms	In the past year, has the participant experienced an inexplicable strong fear in situations such as closed spaces (cave, elevator, airplane, etc), in crowds or public, such that they would avoid such situations? (question in CIDI-SF)

	Neurasthenia	Has the participant ever been told by a doctor that they had neurasthenia (a common diagnosis in China and East Asia, with symptoms including chronic fatigue, headache, dizziness, concentration difficulties, and sleep disturbances)?
	Psychiatric disorder	Has the participant ever been told by a doctor that they had a psychiatric disorder?
	Unsatisfied with life	In general, is the participant "unsatisfied" or "very unsatisfied" with life, compared to "very satisfied", "satisfied", and "neither satisfied nor unsatisfied"?
	Stressful life events	In the past two years, has the participant experienced at least one of the following stressful life events: marital separation/divorce; loss of job/retirement; bankruptcy; violence; family conflict; injury/traffic accident; death/major illness of spouse; death/major illness of close family member; natural disaster; loss of income/living on debt?
	Sleep duration	How many hours does the participant sleep per day (including naps)?
	Sleep problems	In the past month, has the participant experienced at least one of the following sleep problems: having trouble falling asleep (sleep onset latency ≥ 30 min) after going to bed or waking up in the middle of the night at least 3 days a week; waking up too early and not be able to get back to sleep at least 3 days a week; having trouble keeping sober-minded during daytime because of bad sleep at least 3 days a week?
	Snoring	Does the participant snore during sleep?
Physical health	Poor self-rated health	Is the participant's self-rated health "poor", compared to "fair", "good", and "excellent"?
	Worse comparative health	Does the participant rate their general health "worse" than someone of their own age, compared "about the same" and "better"?
	Asthma	Has the participant ever been told by a doctor that they had asthma?
	Chronic obstructive pulmonary disease	Does the participant have a history of COPD (self-reported OR meets the old Global Initiative for Chronic Obstructive Lung Disease [GOLD] stage 1+ criterion)
	Emphysema/ bronchitis	Has the participant ever been told by a doctor that they had emphysema/bronchitis?
	Tuberculosis	Has the participant ever been told by a doctor that they had tuberculosis?
	Rheumatoid arthritis	Has the participant ever been told by a doctor that they had rheumatoid arthritis?
	Rheumatoid heart disease	Has the participant ever been told by a doctor that they had rheumatoid heart disease?
	Chronic heart disease	Has the participant ever been told by a doctor that they had chronic heart disease?

	Stroke/transient ischemic attack	Has the participant ever been told by a doctor that they had stroke/transient ischemic attack?
	Diabetes	Does the participant has a history of diabetes (self-reported OR random blood glucose >= 11.1 mmol/L OR fasting blood glucose >= 7.0 mmol/L)
	Peptic ulcer	Has the participant ever been told by a doctor that they had peptic ulcer?
	Cirrhosis/chronic hepatitis	Has the participant ever been told by a doctor that they had cirrhosis/chronic hepatitis?
	Gallstone/gallbladder disease	Has the participant ever been told by a doctor that they had gallstone/gallbladder disease?
	Kidney disease	Has the participant ever been told by a doctor that they had kidney disease?
	Head injury	Has the participant ever been told by a doctor that they had a head injury?
	Fracture	Has the participant ever been told by a doctor that they had a fracture?
	Cancer	Has the participant ever been told by a doctor that they had cancer?
Clinical measurements	Body mass index	Body mass index calculated from measured standing height and weight (kg/m^2)
	Standing height	Standing height (without shoes; mm)
	Sitting height	Sitting height (mm)
	Systolic blood pressure	Mean systolic blood pressure measurement (of the two taken; mmHg)
	Diastolic blood pressure	Mean diastolic blood pressure measurement (of the two taken; mmHg)
	Heart rate	Mean heart rate measurement (of the two taken; bpm)
	Random glucose	Random blood sugar result from blood test (mmol/L)
Lifestyle	Physical activity	Total daily physical activity (Metabolic Equivalent of Task; MET hours/day)
	Food diversity	Derived by aggregating the intake frequency (Never=0, 1-3 days per week=1, 4-7 days per week=2) across 12 food categories: rice, wheat, other staple food, meat, poultry, fish, eggs, dairy, fresh fruit, fresh vegetables, preserved vegetables, and soybeans.

	Ever regular alcohol drinking	Has the participant ever been a regular alcohol drinker ("weekly", "ex-regular", and "reduced intake" compared to "never regular", "occasional", and "monthly")?
	Current regular alcohol drinking	Is the participant currently a regular alcohol drinker ("weekly" compared to "never regular", "occasional", "monthly", "ex-regular", and "reduced intake")?
	Age of alcohol initiation	At about what age did the participant start drinking some alcohol in most weeks?
	Problem alcohol drinking	Does the participant have problem drinking, defined as having at least one of the following indicators: ever drinking in the morning; being unable to work or to do anything due to drinking; feeling depressed, irritated or losing control after drinking (i.e. negative emotions); being unable to keep away from drinking; and having shakes when stopping drinking?
	Ever regular smoking	Has the participant ever been a regular smoker ("smoker" and "ex-regular smoker" compared to "never smoker" and "occasional smoker")?
	Current regular smoking	Is the participant currently a regular smoker ("smoker" compared to "never smoker", "occasional smoker", and "ex-regular smoker")?
	Age of smoking initiation	At about what age did the participant first start smoking on most days?
	Cigarettes per day	Cigarette equivalents smoked per day
Reproductive history (female only)	Age at menarche	How old was the participant when they had their first menstrual period?
	Post menopause	Has the participant had their menopause ("yes" compared to "currently" and "no)?
	Age at menopause	At what age did the participant finish their menopause?
	Number of pregnancies	How many times has the participant ever been pregnant?
	Number of live births	Number of live births
	Age at first live birth	At what age did the participant have their first live birth?
	Number of still births	Number of still births

	Had hysterectomy	Has the participant had a hysterectomy?
	Had breast lump removed	Has the participant had a breast lump removed?
	Had ovary removed	Has the participant had one or both ovaries removed?

Note. PheWAS: Phenome-wide association study. CKB: China Kadoorie Biobank. CIDI-SF: Composite International Diagnostic Interview Short Form.

Supplementary Table 3. Phenotypic correlations between mental disorders and phenotypes in the UK Biobank.

	BMI	SmkInit	CigDay	CanUD	EA	Income	SCZ	MD
BMI	1.000	0.034	0.092	-0.020	-0.105	-0.095	0.012	0.063
SmkInit	0.034	1.000	0.000	0.122	-0.064	-0.046	0.012	0.038
CigDay	0.092	0.000	1.000	-0.098	-0.077	-0.106	0.073	0.070
CanUD	-0.020	0.122	-0.098	1.000	0.027	0.012	0.010	0.025
EA	-0.105	-0.064	-0.077	0.027	1.000	0.331	-0.001	-0.038
Income	-0.095	-0.046	-0.106	0.012	0.331	1.000	-0.049	-0.120
SCZ	0.012	0.012	0.073	0.010	-0.001	-0.049	1.000	0.064
MD	0.063	0.038	0.070	0.025	-0.038	-0.120	0.064	1.000

Note. Numbers represent Spearman's rank correlation coefficient (*ρ*). Heavy cannabis use (lifetime cannabis use > 100 times) was used as a proxy for CanUD in the UK Biobank. BMI: Body mass index. BMI: Body mass index. SmkInit: Smoking initiation. CigDay: Cigarettes per day. CanUD: Cannabis use disorder. EA: Educational attainment. SCZ: Schizophrenia. MD: Major depression.

Supplementary Table 4. Associations between polygenic scores for schizophrenia/major depression and their corresponding phenotypes in CKB by sex.

Sex	Mental disorder	n (cases)	n (controls)	GWAS source	OR per SD higher PGS (95% CI)	*p*	R2 on liability scale
Female	SCZ	95	45,717	EAS	1.70 (1.38 – 2.11)	8.25×10^{-7}	3.23%
				Multi (EAS + EUR)	1.81 (1.47 – 2.23)	3.29×10^{-8}	4.30%
	MD	621	45,296	EAS	1.12 (1.03 - 1.21)	0.006	0.17%
				Multi (EAS + EUR)	1.26 (1.16 - 1.36)	1.89×10^{-8}	0.69%
Male	SCZ	63	31,273	EAS	1.53 (1.18 – 1.99)	0.001	1.84%
				Multi (EAS + EUR)	1.48 (1.15 - 1.91)	0.003	1.60%
	MD	285	31,119	EAS	1.14 (1.02 - 1.28)	0.025	0.28%
				Multi (EAS + EUR)	1.26 (1.12 - 1.41)	1.42×10^{-4}	0.69%

Note. Cases of mental disorders were identified from the overall dataset, while controls were identified from the population-representative subset. Two types of polygenic scores were tested for each mental disorder, one trained based on GWAS in EAS and one based on GWAS in both EAS and EUR. SCZ: Schizophrenia. MD: Major depression. GWAS: Genome-wide association studies. EAS: East Asian ancestry population. EUR: European ancestry population. OR: Odds ratio per standard deviation higher in polygenic scores. CI: Confidence interval.

Supplementary Table 5. Within-ancestry genetic correlations between mental disorders and other phenotypes.

Mental disorder	Phenotype	Ancestry	r_g	SE	Z	p
SCZ	MD	EAS	0.41	0.09	4.35	**1.33E-05**
		EUR	0.36	0.02	21.57	**3.30E-103**
	BMI	EAS	-0.05	0.03	-1.46	1.44E-01
		EUR	-0.11	0.01	-7.25	**4.20E-13**
	Height	EAS	-0.04	0.02	-1.70	9.00E-02
		EUR	-0.01	0.01	-0.77	4.40E-01
	EA	EAS	0.14	0.04	3.41	**6.00E-04**
		EUR	0.03	0.02	1.96	**4.96E-02**
	SmkInit	EAS	-0.10	0.04	-2.83	**4.70E-03**
		EUR	0.17	0.02	8.87	**7.01E-19**
	CigDay	EAS	0.09	0.05	1.63	1.03E-01
		EUR	0.10	0.02	4.36	**1.28E-05**
MD	SCZ	EAS	0.41	0.09	4.35	**1.33E-05**
		EUR	0.36	0.02	21.57	**3.30E-103**
	BMI	EAS	-0.19	0.07	-2.63	**8.60E-03**
		EUR	0.17	0.02	10.84	**2.16E-27**
	SmkInit	EAS	0.00	0.08	-0.01	9.90E-01
		EUR	0.38	0.02	22.43	**1.95E-111**
	CigDay	EAS	0.02	0.13	0.17	8.63E-01
		EUR	0.28	0.03	9.44	**3.67E-21**
	Cataract	EAS	-0.10	0.18	-0.58	5.62E-01
		EUR	0.11	0.05	2.33	**1.96E-02**
	Diabetes	EAS	-0.08	0.06	-1.35	1.77E-01
		EUR	0.06	0.02	3.36	**8.00E-04**
	Stroke	EAS	0.05	0.13	0.35	7.27E-01
		EUR	0.19	0.03	6.54	**6.24E-11**
	CAD	EAS	-0.04	0.10	-0.39	6.96E-01
		EUR	0.25	0.02	13.94	**3.69E-44**
	Lung function	EAS	0.15	0.25	0.59	5.57E-01
		EUR	0.00	0.01	0.44	6.60E-01
	Asthma	EAS	-0.18	0.10	-1.80	7.18E-02
		EUR	0.38	0.02	17.80	**7.61E-71**
	RA	EAS	-0.03	0.10	-0.33	7.41E-01
		EUR	0.04	0.02	1.77	7.74E-02
	PUD	EAS	0.11	0.12	0.92	3.60E-01
		EUR	0.16	0.03	4.98	**6.51E-07**
	Gallstone	EAS	0.21	0.15	1.41	1.58E-01
		EUR	0.29	0.03	10.52	**6.63E-26**

Note. Mental disorder-phenotype pairs that were significant in the phenome-wide association analysis were tested here. Only phenotypes with publicly available GWAS in both EAS and EUR were included. SCZ: Schizophrenia. MD: Major depression. EAS: East Asian ancestry population. EUR: European ancestry population. BMI: Body mass index. SmkInit: Smoking initiation. CigDay: Cigarettes

per day. CAD: Coronary artery disease. RA: Rheumatoid arthritis. PUD: Peptic ulcer disease. SE: Standard error.

Supplementary Table 6. Cross-ancestry genetic correlations between mental disorders and other phenotypes.

Mental disorder (1)	Ancestry (1)	Phenotype (1)	Ancestry (2)	ρ_{ge}	SE	Z	p
SCZ	EAS	MD	EUR	0.22	0.06	3.84	**1.24E-04**
		BMI		-0.22	0.06	-3.51	**4.44E-04**
		Height		0.02	0.04	0.64	5.22E-01
		EA		0.15	0.06	2.55	**1.09E-02**
		SmkInit		0.00	0.07	-0.03	9.76E-01
		CigDay		-0.01	0.06	-0.19	8.47E-01
	EUR	MD	EAS	0.32	0.14	2.25	**2.43E-02**
		BMI		-0.12	0.04	-2.63	**8.59E-03**
		Height		-0.05	0.03	-1.36	1.73E-01
		EA		0.08	0.05	1.63	1.04E-01
		SmkInit		0.05	0.05	0.94	3.45E-01
		CigDay		0.05	0.06	0.78	4.33E-01
MD	EAS	SCZ	EUR	0.22	0.06	3.84	**1.24E-04**
		BMI		-0.25	0.10	-2.48	**1.33E-02**
		SmkInit		-0.01	0.10	-0.09	9.27E-01
		CigDay		-0.13	0.13	-0.98	3.27E-01
		Cataract		-0.14	0.26	-0.53	5.98E-01
		Diabetes		-0.01	0.10	-0.09	9.26E-01
		Stroke		-0.28	0.23	-1.22	2.22E-01
		CAD		-0.16	0.15	-1.06	2.88E-01
		Lung function		-0.08	0.06	-1.25	2.12E-01
		Asthma		-0.07	0.19	-0.37	7.08E-01
		RA		NA	NA	NA	NA
		PUD		0.04	0.20	0.21	8.34E-01
		Gallstone		0.13	0.14	0.91	3.64E-01
	EUR	SCZ	EAS	0.32	0.14	2.25	**2.43E-02**
		BMI		0.02	0.04	0.56	5.78E-01
		SmkInit		0.30	0.04	6.86	**6.87E-12**
		CigDay		0.24	0.05	5.19	**2.05E-07**
		Cataract		0.10	0.07	1.40	1.60E-01
		Diabetes		0.07	0.03	2.35	**1.88E-02**
		Stroke		0.07	0.06	1.08	2.81E-01
		CAD		0.06	0.04	1.47	1.43E-01
		Lung function		-0.09	0.11	-0.81	4.18E-01
		Asthma		0.10	0.05	1.97	**4.89E-02**
		RA		-0.05	0.05	-1.07	2.84E-01
		PUD		0.16	0.06	2.79	**5.27E-03**
		Gallstone		0.12	0.07	1.68	9.25E-02

Note. Mental disorder-phenotype pairs that were significant in the phenome-wide association analysis were tested here. Only phenotypes with publicly available GWAS in both EAS and EUR were included. Mental disorders (1) in one ancestry (1) were tested against phenotypes (2) in the other ancestry (2). NA represents unreliable estimates that had SE > 0.3. SCZ: Schizophrenia. MD: Major depression. EAS: East Asian ancestry population. EUR: European ancestry population. BMI: Body mass index. SmkInit: Smoking initiation. CigDay: Cigarettes per day. CAD: Coronary artery disease. RA: Rheumatoid arthritis. PUD: Peptic ulcer disease. SE: Standard error.

Supplementary Figure 1. Selection of cases and controls in PheWAS.

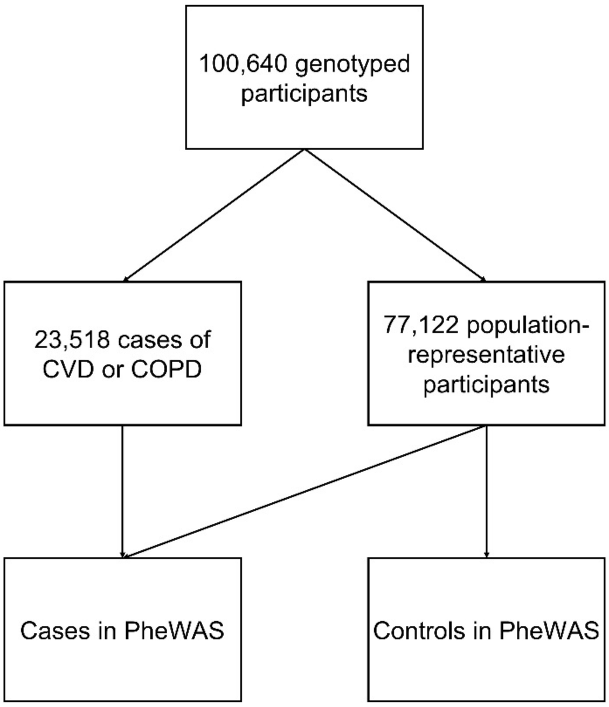

Note. In PheWAS, cases of all disease-related phenotypes were identified from the overall dataset (including both selected CVD/COPD cases and population-representative participants), while controls were only identified from the population-representative subset. CVD: Cardiovascular disease. COPD: Chronic obstructive pulmonary disease.

Supplementary Figure 2. Performance of schizophrenia polygenic scores in CKB.

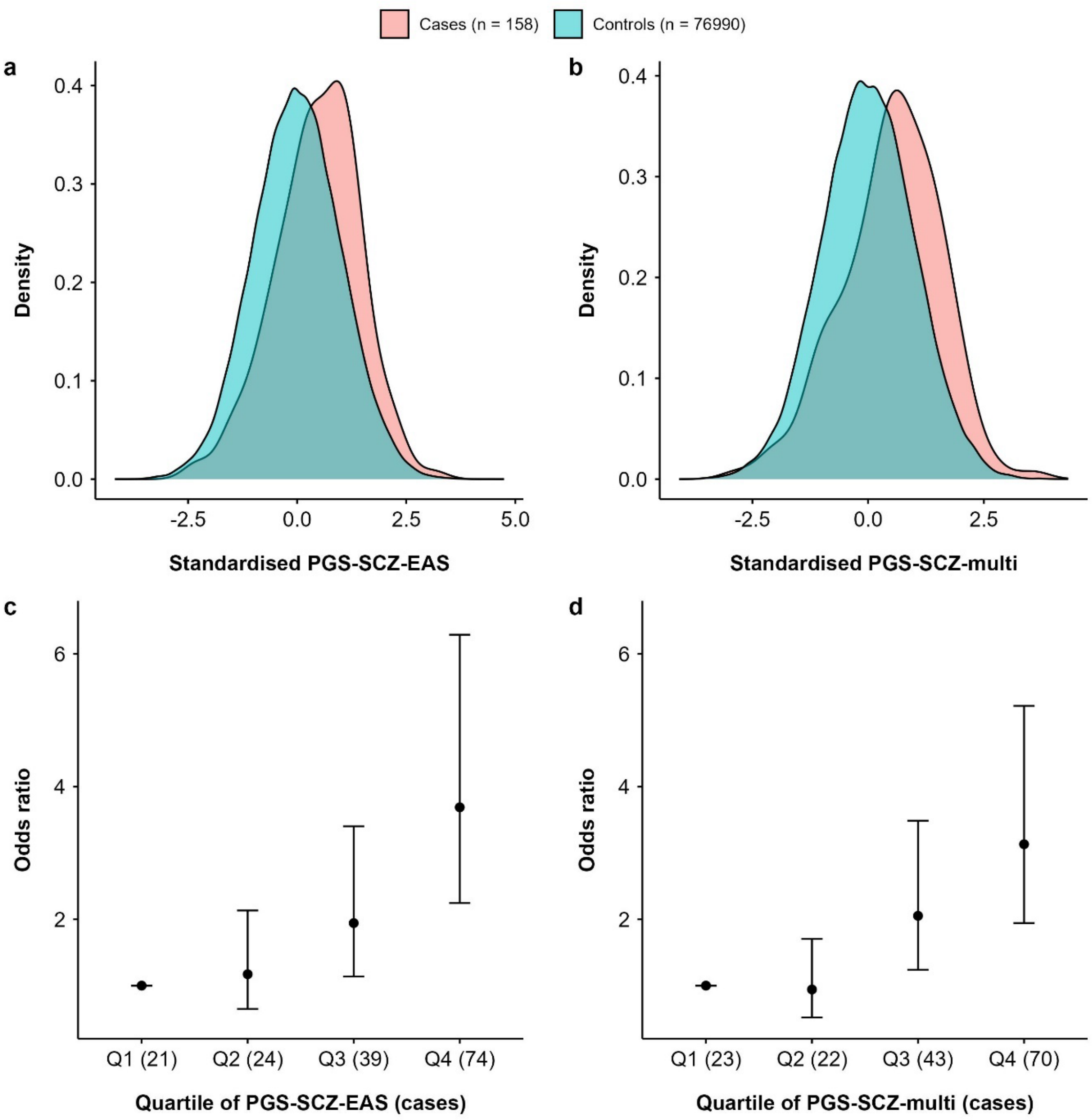

Note. a, Distribution of PGS-SCZ-EAS. b, Distribution of PGS-SCZ-multi. c, Associations between PGS-SCZ-EAS and SCZ by quartile. d, Associations between PGS-SCZ-multi and SCZ by PGS quartile. Cases of SCZ were identified from the overall dataset, while controls were identified from the population-representative subset. Two types of PGS tested, one based on GWAS in EAS and one based on GWAS in both EAS and EUR. PGS: Polygenic score. SCZ: Schizophrenia. EAS: East Asian ancestry population. EUR: European ancestry population.

Supplementary Figure 3. Performance of major depression polygenic scores in CKB.

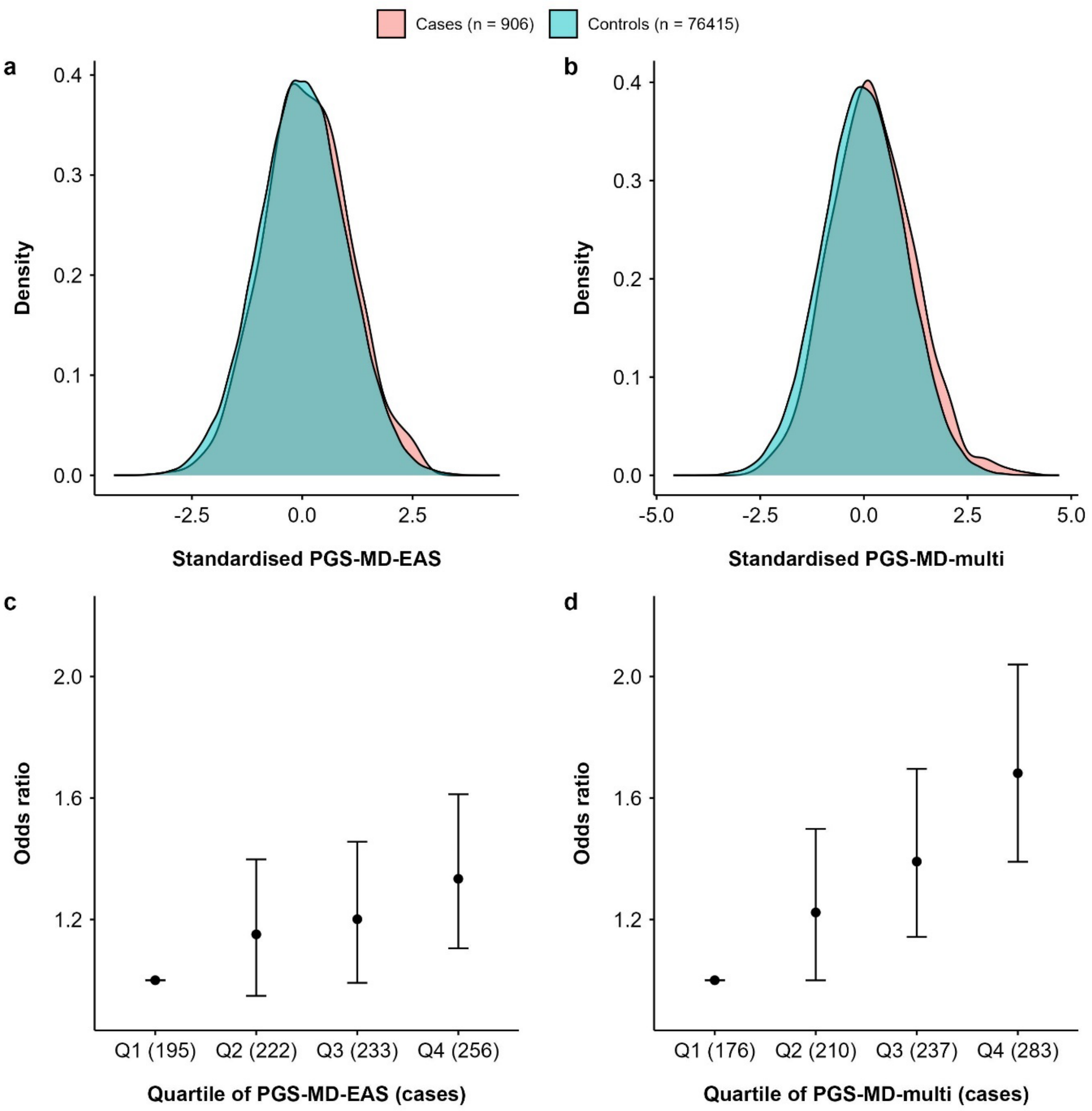

Note. a, Distribution of PGS-MD-EAS. b, Distribution of PGS-MD-multi. c, Associations between PGS-MD-EAS and MD by quartile. d, Associations between PGS-MD-multi and MD by PGS quartile. Cases of MD were identified from the overall dataset, while controls were identified from the population-representative subset. Two types of PGS tested, one based on GWAS in EAS and one based on GWAS in both EAS and EUR. PGS: Polygenic score. MD: Major depression. EAS: East Asian ancestry population. EUR: European ancestry population.

Supplementary Figure 4. Performance of schizophrenia polygenic scores among females in CKB.

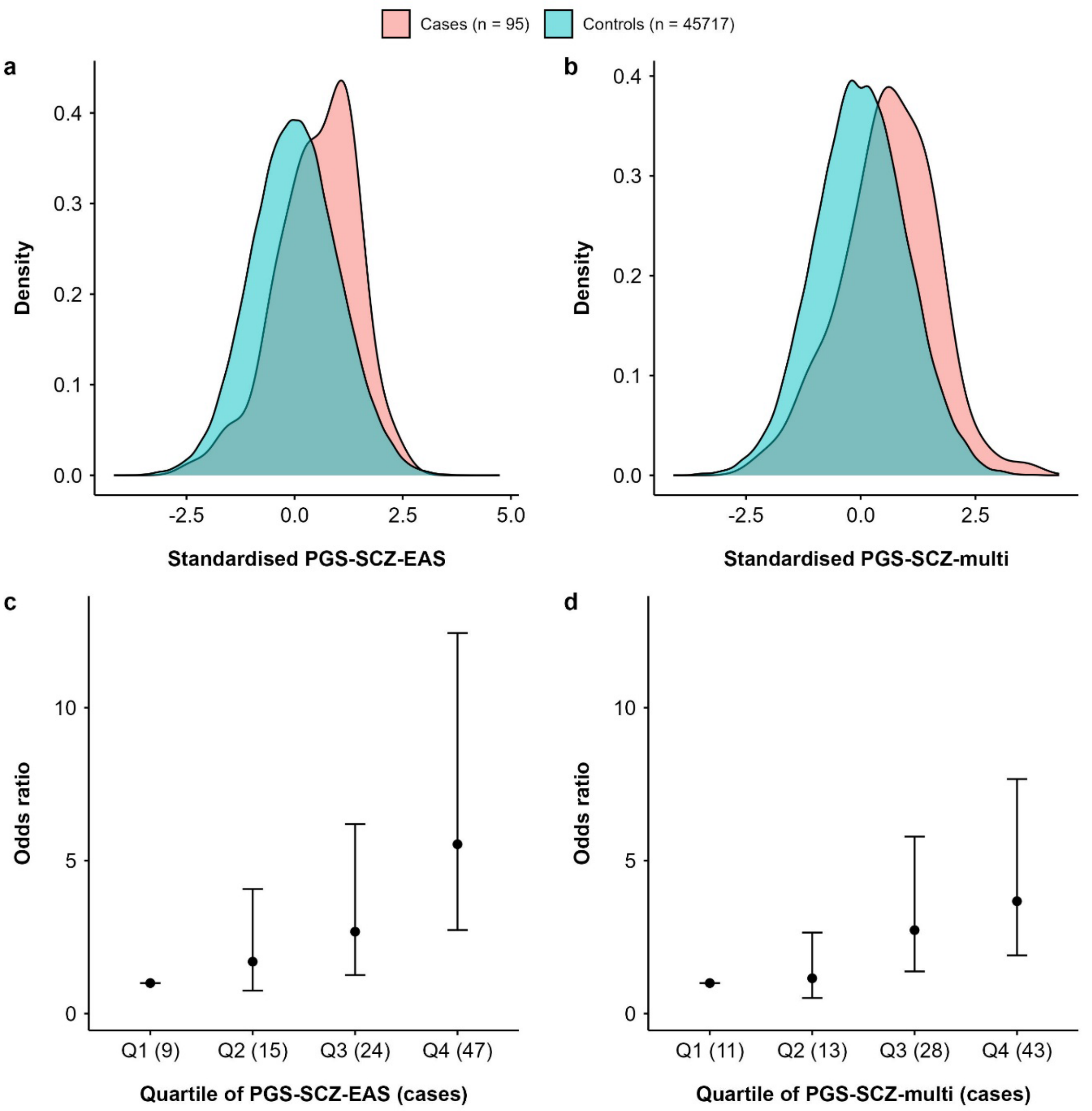

Note. a, Distribution of PGS-SCZ-EAS. b, Distribution of PGS-SCZ-multi. c, Associations between PGS-SCZ-EAS and SCZ by quartile. d, Associations between PGS-SCZ-multi and SCZ by PGS quartile. Cases of SCZ were identified from the overall dataset, while controls were identified from the population-representative subset. Two types of PGS tested, one based on GWAS in EAS and one based on GWAS in both EAS and EUR. PGS: Polygenic score. SCZ: Schizophrenia. EAS: East Asian ancestry population. EUR: European ancestry population.

Supplementary Figure 5. Performance of major depression polygenic scores among females in CKB.

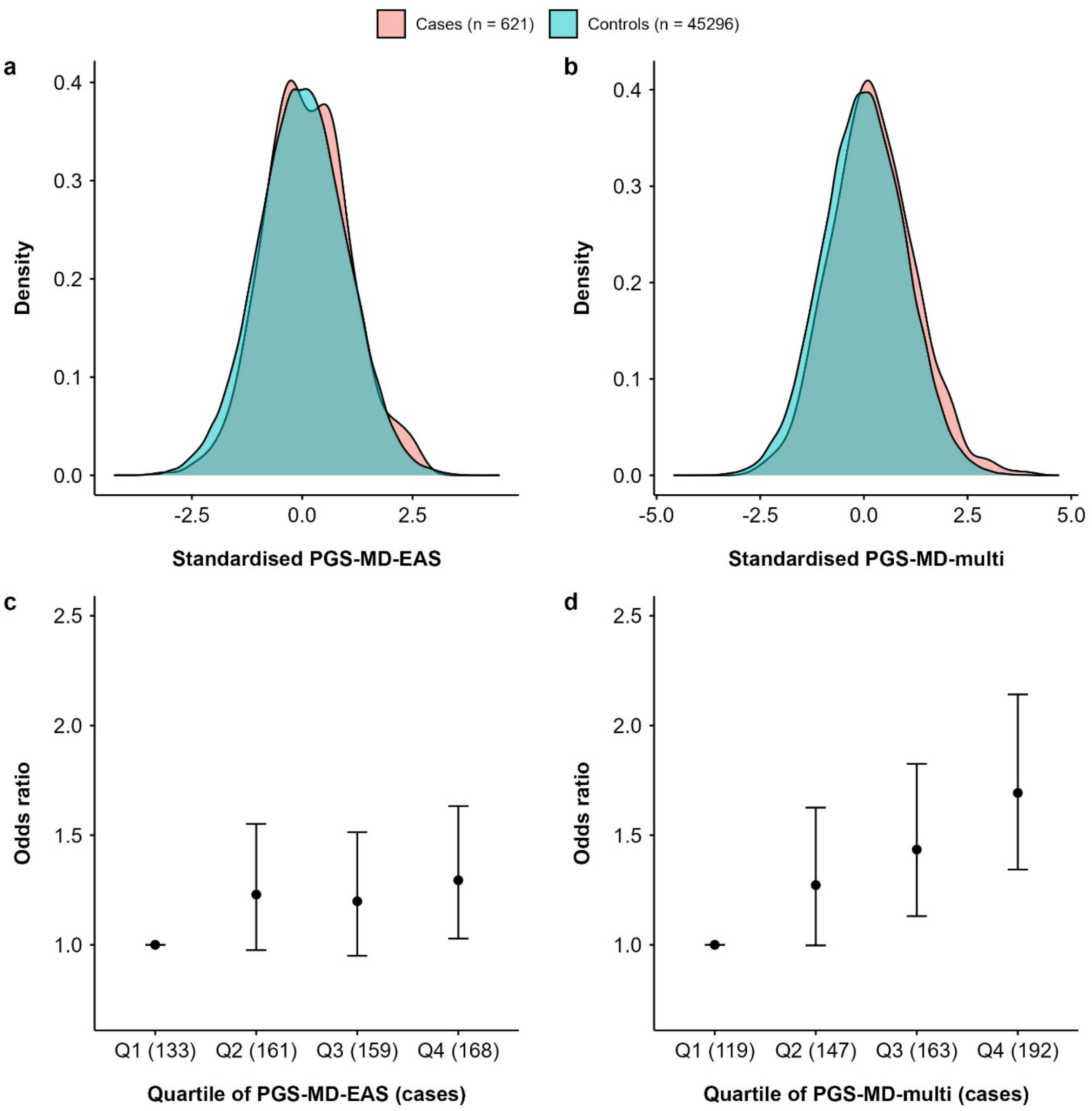

Note. a, Distribution of PGS-MD-EAS. b, Distribution of PGS-MD-multi. c, Associations between PGS-MD-EAS and MD by quartile. d, Associations between PGS-MD-multi and MD by PGS quartile. Cases of MD were identified from the overall dataset, while controls were identified from the population-representative subset. Two types of PGS tested, one based on GWAS in EAS and one based on GWAS in both EAS and EUR. PGS: Polygenic score. MD: Major depression. EAS: East Asian ancestry population. EUR: European ancestry population.

Supplementary Figure 6. Performance of schizophrenia polygenic scores among males in CKB.

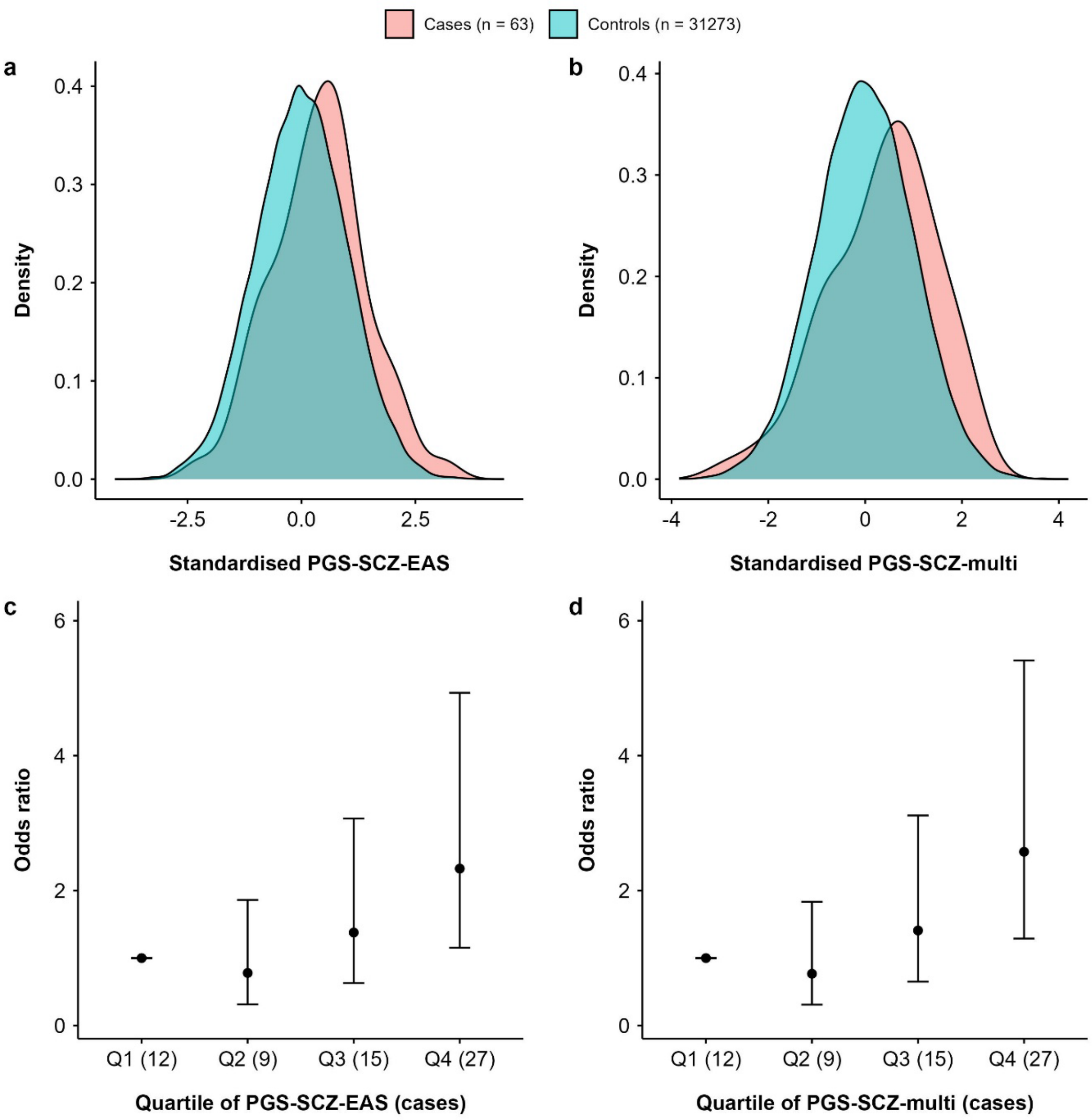

Note. a, Distribution of PGS-SCZ-EAS. b, Distribution of PGS-SCZ-multi. c, Associations between PGS-SCZ-EAS and SCZ by quartile. d, Associations between PGS-SCZ-multi and SCZ by PGS quartile. Cases of SCZ were identified from the overall dataset, while controls were identified from the population-representative subset. Two types of PGS tested, one based on GWAS in EAS and one based on GWAS in both EAS and EUR. PGS: Polygenic score. SCZ: Schizophrenia. EAS: East Asian ancestry population. EUR: European ancestry population.

Supplementary Figure 7. Performance of major depression polygenic scores among males in CKB.

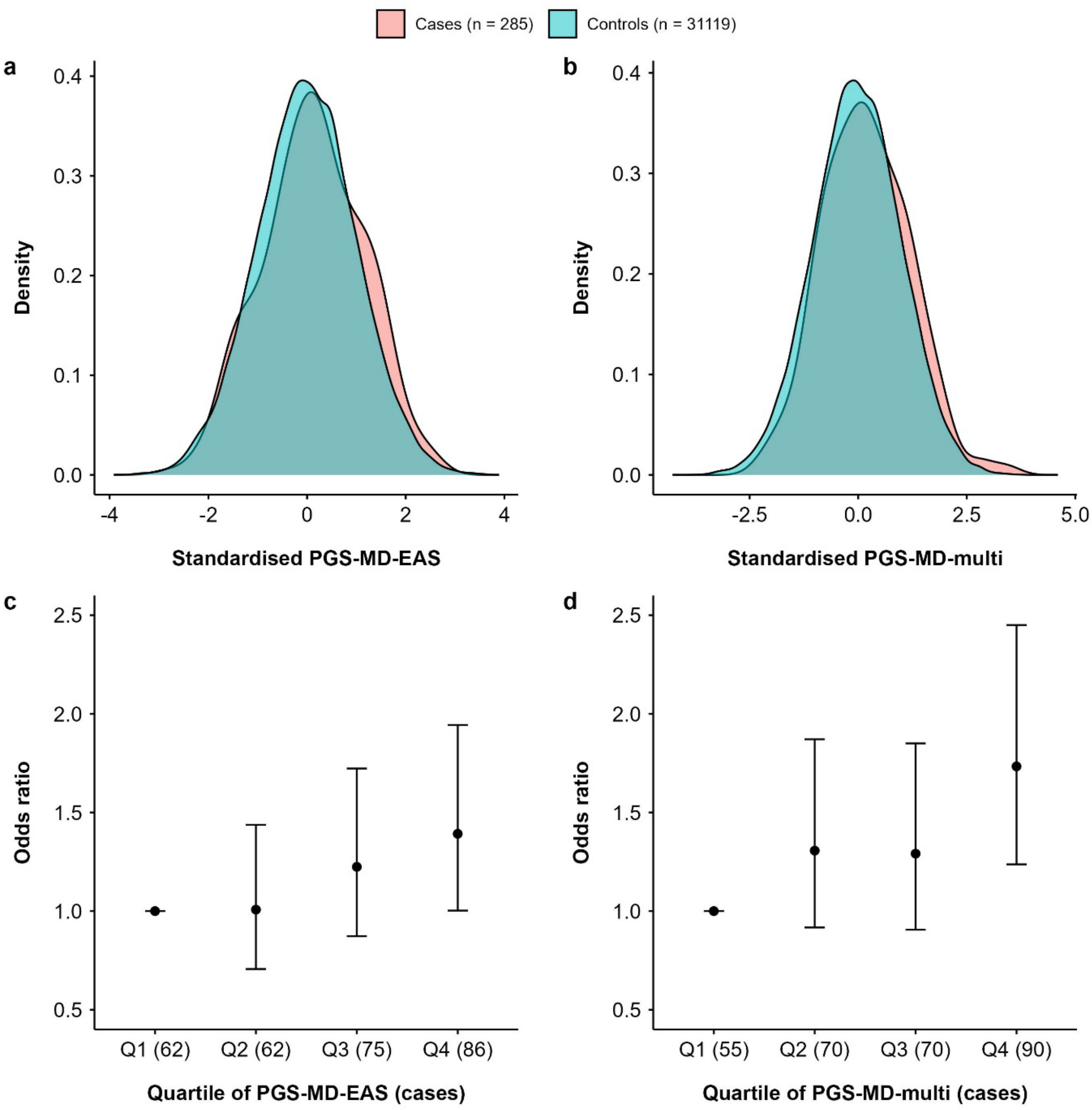

Note. a, Distribution of PGS-MD-EAS. b, Distribution of PGS-MD-multi. c, Associations between PGS-MD-EAS and MD by quartile. d, Associations between PGS-MD-multi and MD by PGS quartile. Cases of MD were identified from the overall dataset, while controls were identified from the population-representative subset. Two types of PGS tested, one based on GWAS in EAS and one based on GWAS in both EAS and EUR. PGS: Polygenic score. MD: Major depression. EAS: East Asian ancestry population. EUR: European ancestry population.

Supplementary Figure 8. Phenome-wide associations with polygenic scores for schizophrenia and major depression among females in CKB.

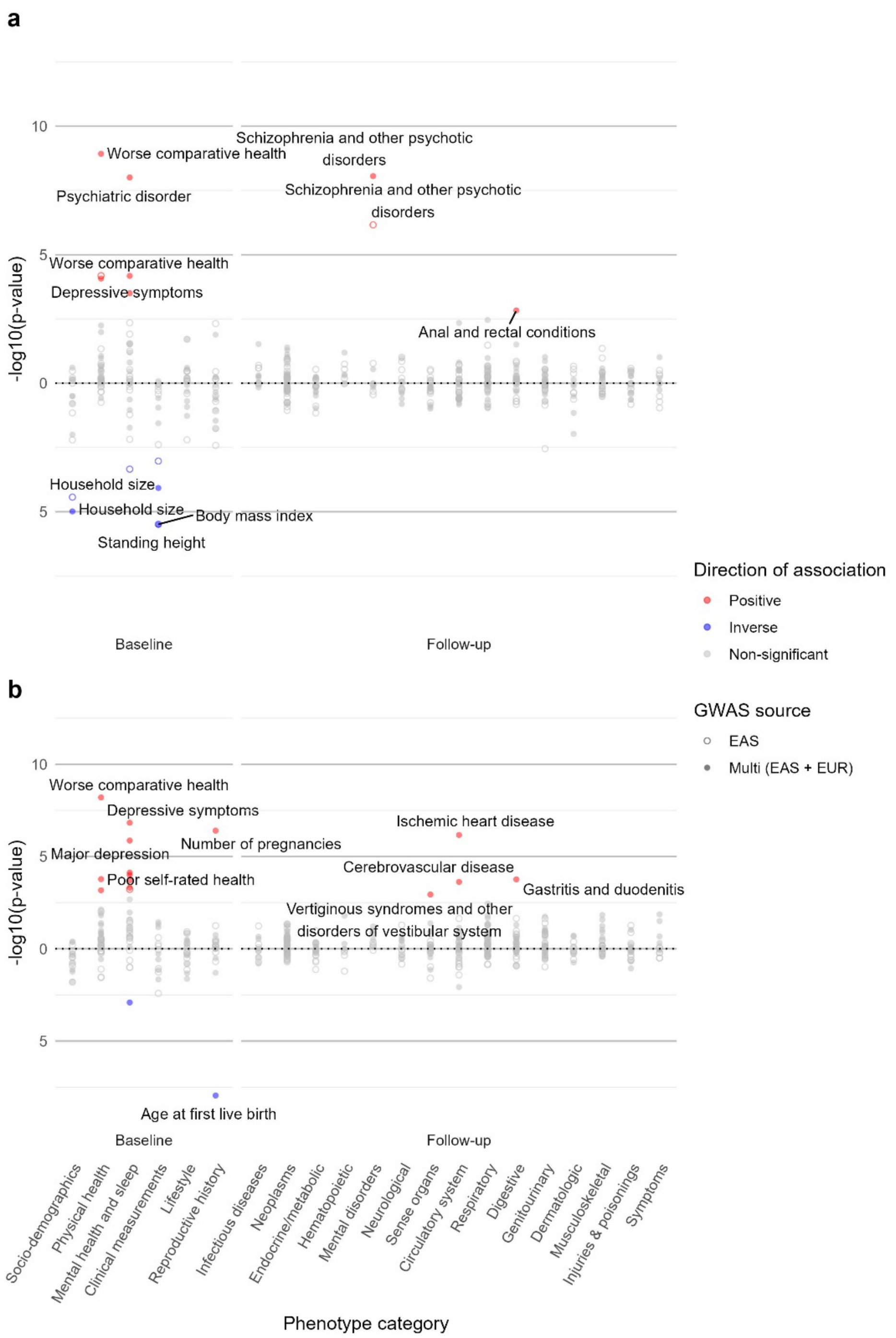

Note. a, Results of polygenic scores for schizophrenia. b, Results of polygenic scores for major depression. A total of 250 phenotypes (67 at baseline and 183 at follow-up) were tested. The shape of dots indicates the GWAS source, while the colour of dots indicates the direction of association. Results were corrected for multiple testing with a false discovery rate = 0.05. The top two most significant associations in each phenotype category are labelled. PGS: Polygenic score. SCZ: Schizophrenia. MD: Major depression. GWAS: Genome-wide association studies. EAS: East Asian ancestry population. EUR: European ancestry population.

Supplementary Figure 9. Phenome-wide associations with polygenic scores for schizophrenia and major depression among males in CKB.

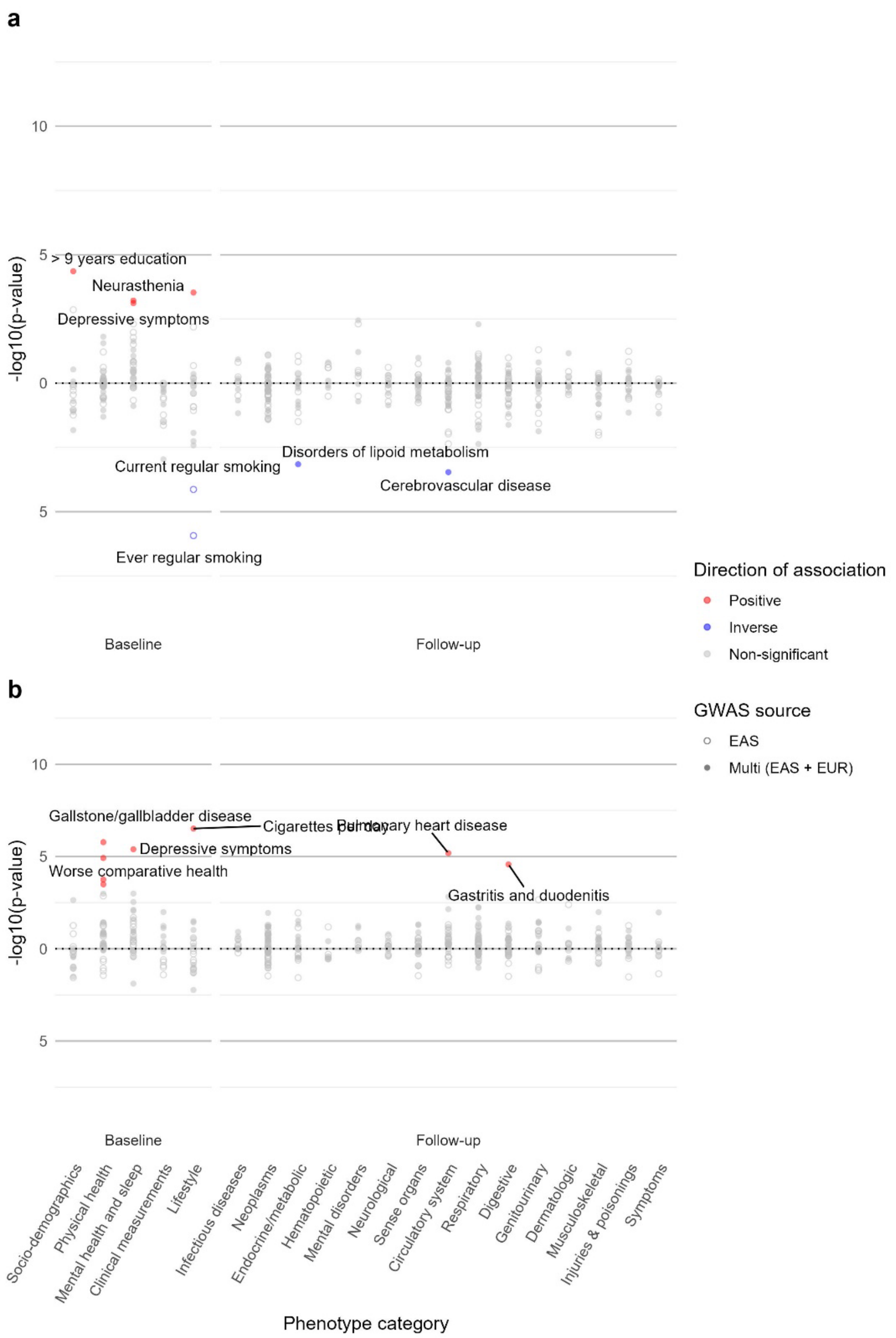

Note. a, Results of polygenic scores for schizophrenia. b, Results of polygenic scores for major depression. A total of 232 phenotypes (57 at baseline and 175 at follow-up) were tested. The shape of dots indicates the GWAS source, while the colour of dots indicates the direction of association. Results were corrected for multiple testing with a false discovery rate = 0.05. The top two most significant associations in each phenotype category are labelled. PGS: Polygenic score. SCZ: Schizophrenia. MD: Major depression. GWAS: Genome-wide association studies. EAS: East Asian ancestry population. EUR: European ancestry population.

Supplementary Figure 10. Cross-ancestry genetic correlations between schizophrenia/major depression and related phenotypes.

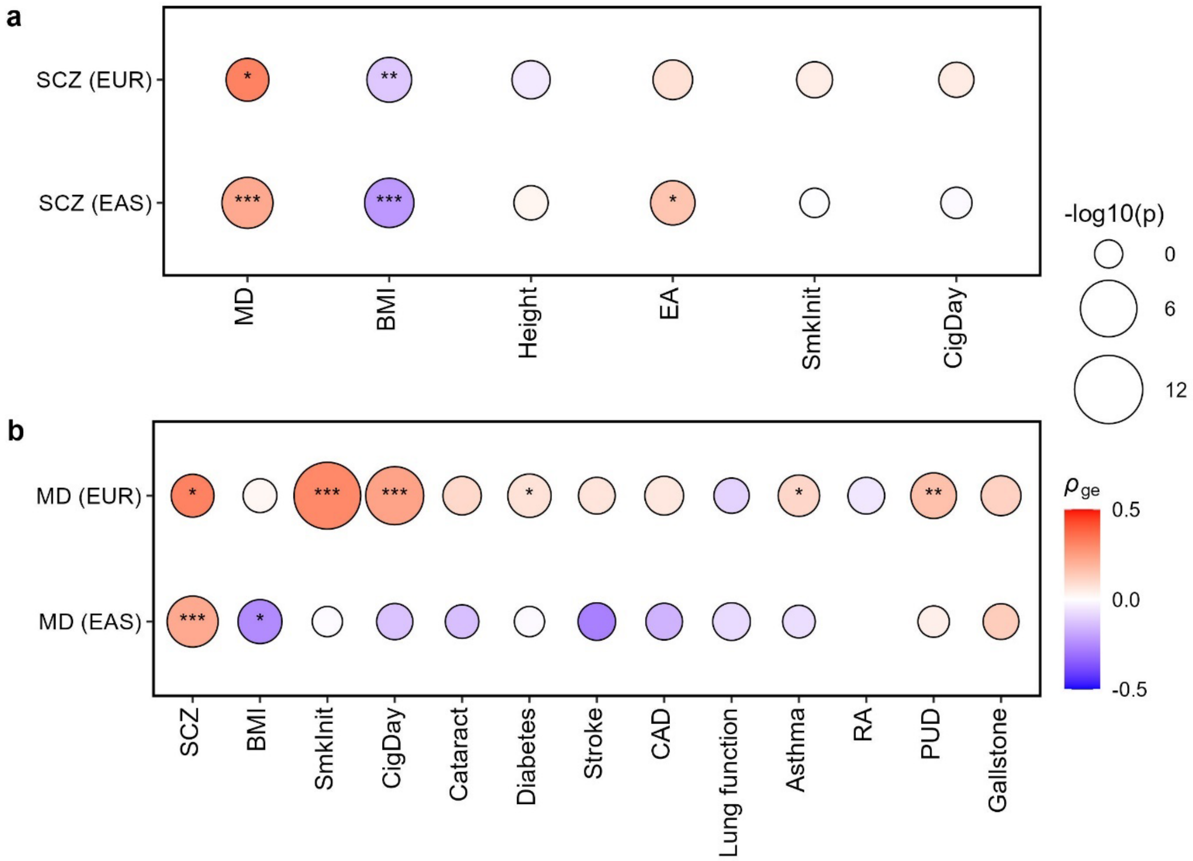

Note. a, Genetic correlations with SCZ. b, Genetic correlations with MD. Cross-ancestry genetic effect correlations (ρ_{ge}) were tested by Popcorn, using GWAS on SCZ/MD in one ancestry and GWAS on phenotypes in the other ancestry. Mental disorder-phenotype pairs that were significant in the phenome-wide association analysis were tested here. Only phenotypes with publicly available GWAS in both EAS and EUR were included. The colour of the circles indicates the correlation coefficient. The size of the circles is scaled to -log10(p-value). *: $p < 0.05$; **: $p < 0.01$; ***: $p < 0.001$. Only estimates with SE < 0.3 are shown here. SCZ: Schizophrenia. MD: Major depression. EAS: East Asian ancestry population. EUR: European ancestry population. BMI: Body mass index. SmkInit: Smoking initiation. CigDay: Cigarettes per day. CAD: Coronary artery disease. RA: Rheumatoid arthritis. PUD: Peptic ulcer disease. SE: Standard error.

Supplementary Figure 11. Bi-directional Mendelian Randomisation between schizophrenia/major depression and other phenotypes with a more stringent threshold.

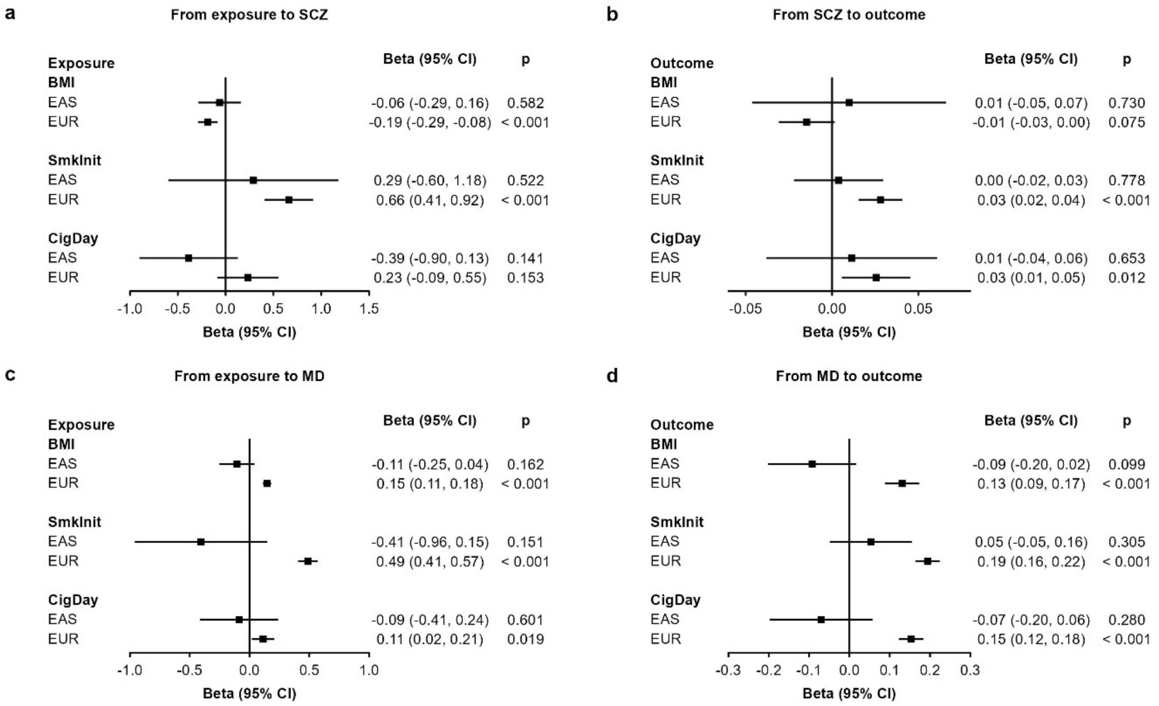

Note. a, From exposure to SCZ. b, From SCZ to outcome. c, From exposure to MD. d, From MD to outcome. Results shown here are based on estimates from the Wald ratio test (MD as exposure) and the inverse variance weighted method (all other exposures). Genetic instruments were selected based on $p < 5 \times 10^{-8}$ in both EAS and EUR after clumping. SCZ: Schizophrenia. MD: Major depression. EAS: East Asian ancestry population. EUR: European ancestry population. BMI: Body mass index. SmkInit: Smoking initiation. CigDay: Cigarettes per day. CI: Confidence interval.
